# *Agrobacterium*: nature’s genetic engineer

**DOI:** 10.3389/fpls.2014.00730

**Published:** 2015-01-06

**Authors:** Eugene W. Nester

**Affiliations:** Department of Microbiology, University of Washington, Seattle, WA, USA

**Keywords:** *Agrobacterium*, crown gall, plant genetic engineering, plant disease, *Agrobacterium*-mediated transformation

## Abstract

*Agrobacterium* was identified as the agent causing the plant tumor, crown gall over 100 years ago. Since then, studies have resulted in many surprising observations. Armin Braun demonstrated that *Agrobacterium* infected cells had unusual nutritional properties, and that the bacterium was necessary to start the infection but not for continued tumor development. He developed the concept of a tumor inducing principle (TIP), the factor that actually caused the disease. Thirty years later the TIP was shown to be a piece of a tumor inducing (Ti) plasmid excised by an endonuclease. In the next 20 years, most of the key features of the disease were described. The single-strand DNA (T-DNA) with the endonuclease attached is transferred through a type IV secretion system into the host cell where it is likely coated and protected from nucleases by a bacterial secreted protein to form the T-complex. A nuclear localization signal in the endonuclease guides the transferred strand (T-strand), into the nucleus where it is integrated randomly into the host chromosome. Other secreted proteins likely aid in uncoating the T-complex. The T-DNA encodes enzymes of auxin, cytokinin, and opine synthesis, the latter a food source for *Agrobacterium*. The genes associated with T-strand formation and transfer (*vir*) map to the Ti plasmid and are only expressed when the bacteria are in close association with a plant. Plant signals are recognized by a two-component regulatory system which activates *vir* genes. Chromosomal genes with pleiotropic functions also play important roles in plant transformation. The data now explain Braun’s old observations and also explain why *Agrobacterium* is nature’s genetic engineer. Any DNA inserted between the border sequences which define the T-DNA will be transferred and integrated into host cells. Thus, *Agrobacterium* has become the major vector in plant genetic engineering.

## INTRODUCTION

*Agrobacterium* is a truly remarkable organism. Its study over the last 100 years has revolutionized plant molecular genetics, and has given birth to a whole new industry dedicated to the genetic modification of plants. Initially, studies were aimed solely at identifying the cause of destructive galls on ornamental plants and fruit trees. In the United States, two plant pathologists, Smith and Townsend (1907) reported that the causative agent of the disease, crown gall, was a bacterium that they named *Bacterium tumefaciens*. More than 30 years later, Armin Braun, a scientist at the Rockefeller Institute in Princeton, New Jersey demonstrated that this was a very unusual plant disease with properties never seen before. His observations raised several intriguing questions. How could a bacterium cause a disease that changed the nutritional properties of the infected cells (Braun, 1958)? And most surprisingly, how could these changes occur in the absence of the bacterium (White and Braun, 1941)? To answer these questions required technologies not available to Braun. In the 1960s, a number of laboratories skilled in the techniques of bacterial genetics and nucleic acid chemistry began to study the system. In a relatively short time, several key discoveries were made. An unusually large plasmid was discovered and its association with gall formation demonstrated (Zaenen et al., 1974). This was followed by the discovery that a piece of the plasmid was transferred and randomly integrated into the chromosome of the plant cell (Chilton et al., 1977; Lemmers et al., 1980; Thomashow et al., 1980; Zambryski et al., 1980). Over the next 10 years, studies from laboratories around the world answered these major questions. What do the genes transferred to the plant cell encode? What signals are exchanged between plants and bacteria? And why has *Agrobacterium* developed the complex machinery required to form tumors on plants? The answers to many of these questions have resulted in several paradigms of general biological importance which relate not only to bacterial–plant interactions but also to bacterial-animal interactions. An understanding of the basic biology of this unique system made possible the development of *Agrobacterium* as the key player in the genetic modification of plants. However, this bacterium has capabilities that extend beyond plant cell transformation. In the laboratory, *Agrobacterium* can transfer its T-DNA into representative algae (Kumar et al., 2004), fungi (Bundock et al., 1995), and even human cells (Kunik et al., 2001). Thus, what *Agrobacterium* has made possible in plant cell studies should now be possible in the study of other eukaryotic cells.

The story of crown gall must acknowledge the features of *Agrobacterium* which have contributed to the rapid progress made in understanding this system. The organism grows rapidly on a simple medium, is amenable to genetic manipulations developed in *Escherichia coli* and the assays for gene transfer are inexpensive and rapid. Further, it has a relatively small genome that lent itself to sequencing and genome analysis long before sequencing became routine (Goodner et al., 2001; Wood et al., 2001). Those in the field of agrobiology know it’s an organism which is a pleasure to study!

## THE EARLY YEARS

In 1907, two American plant pathologists, Erwin Smith and Charles Townsend reported that the agent causing the common, destructive disease of a variety of ornamental plants called crown gall was a bacterium (Smith and Townsend, 1907). The person most responsible for bridging the gap between the description of the pathogen and the modern era of crown gall research was Armin Braun. His seminal contributions over 35 years, beginning in the 1940s, set the stage for the molecular analysis beginning in the 1960s (Binns, 2005). Braun observed how unique this disease was. He demonstrated that although living bacteria were necessary to start the infection, once initiated, the tumor developed in their absence (White and Braun, 1941). Further, Braun (1958) discovered that tumor cells could be cultured in media lacking the plant hormones, auxin and cytokinin, which are necessary for growth of normal cells. He recognized that some product of *Agrobacterium*, not the bacteria themselves, was altering the properties of plant cells. He developed the concept that this product was the actual tumor inducing principle or TIP (Braun, 1947; Braun and Mandel, 1948). These observations were very important because they provided valuable clues as to the mechanism by which *Agrobacterium* transforms plant cells. It’s interesting that Braun, 1947 suggested that the TIP might be DNA. However, the proof of his prescient suggestion did not come until 30 years later and Braun himself was reluctant to accept this conclusion until he saw convincing data (Binns, 2005).

Important observations continued to be made in a number of laboratories in many countries over the next 20 years. However, it wasn’t until bacterial and molecular genetics became part of the routine thinking of scientists that the modern era of crown gall research became possible. Further, new techniques associated with molecular genetics and nucleic acids had to be developed in order to carry out the experiments which identified and characterized the TIP.

A recent historical, well researched and informative review on *Agrobacterium* covers some of the same ground as the present review (Kado, 2014). Dr. Kado has been a pioneer in the study of *Agrobacterium* and has made numerous important contributions over his many years of research.

## THE MOLECULAR ERA BEGINS

Three papers published in the years 1969–1971 strongly suggested that the TIP was most likely DNA that was transferred from *Agrobacterium* into plant cells. In 1969, the Australian Allen Kerr reported that virulence could be transferred between bacteria when a virulent strain of *Agrobacterium* was inoculated onto tomato plants and several weeks later an avirulent strain was inoculated onto the developing tumor. Using appropriate genetic markers, he showed that virulence was transferred from the virulent to the avirulent strain (Kerr, 1969). However, the mechanism of transfer was not indicated although Kerr commented that DNA transformation of *Agrobacterium* and *Rhizobium* had been reported. In the following year, the laboratory of George Morel in France demonstrated that tumors elicited by different strains of *Agrobacterium* contained low molecular weight compounds not found in normal plant tissues (Petit et al., 1970). The two compounds this laboratory identified were the secondary amines, octopine and nopaline given the general name, opine. Octopine is a condensation product of arginine and pyruvic acid and nopaline a condensation product of arginine and alpha-ketoglutaric acid. Years earlier, another opine, lysopine, a condensation product of lysine and pyruvic acid, had been identified in tumors (Lioret, 1957). The authors suggested that tumors acquired new genetic information from the bacterium. Interestingly, in all three cases, the strain of *Agrobacterium* that induced the synthesis of a particular opine had the ability to degrade the same opine. Unfortunately, several other independent studies, which are likely incorrect, reported that these opines were present in normal plant tissue (Seitz and Hochster, 1964; Johnson et al., 1974). Nevertheless, they clouded the significance of Morel’s observations. The last paper in this set suggesting that DNA was the TIP came from the laboratory of Robert Hamilton at Pennsylvania State University in the USA (Hamilton and Fall, 1971). He observed that cells of strain C58 of *Agrobacterium* when incubated at the elevated temperature of 36°C gave rise to a high proportion of cells that were stably avirulent. Hamilton suggested that heating resulted in the loss of a virulence factor. Since it was known that heating at an elevated temperature could cure strains of their plasmids and plasmids could be transferred, both Kerr and Hamilton looked for evidence of plasmids to explain their respective data but their results were equivocal (Hamilton, 2005; Kerr, 2005).

Being aware of these papers, Bruce Watson, a graduate student of Milton Gordon in Seattle examined strain C58 for plasmids. Despite his concerted efforts, he was unable to demonstrate that this strain contained any (Watson and Nester, 2005).

### THE BREAKTHROUGH

The study which initiated the molecular analysis of crown gall tumor formation came in a report of Ivo Zaenen, a graduate student of Jeff Schell in Belgium. Using alkaline sucrose gradients of bacterial cell lysates, he observed megaplasmids ranging in size from 96 to 156 MDa in 11 virulent strains and none in eight avirulent strains of *Agrobacterium* (Zaenen et al., 1974). Plasmids of such large size had not been reported before. Very quickly, the Belgian group led by Schell and Marc Van Montagu and the Seattle group of Mary-Dell Chilton, Milton Gordon, and Eugene Nester demonstrated that the virulent strain contained a megaplasmid that was not present in the avirulent, heat treated C58 strain (Van Larabeke et al., 1975; Watson et al., 1975). Further, transferring this megaplasmid to the avirulent strain converted it to virulence. The Belgian group termed this plasmid the tumor inducing or Ti plasmid.

### GENOME ORGANIZATION

Before continuing the crown gall saga, it is necessary to point out another important feature of many strains of *Agrobacterium*, its unusual genome organization. The genome of *Agrobacterium tumefaciens* C58 consists of four replicons (Allardet-Servent et al., 1993). These are a circular chromosome, a linear chromosome and two megaplasmids of similar size, pTiC58 and pAtC58. Linear chromosomes are very rare in prokaryotes and even in *Agrobacterium* only biovar I strains (classified on the basis of phenotypic characteristics) contain a linear chromosome.

The C58 genome was sequenced independently by two groups in the United States by a collaborative effort of two academic institutions collaborating with two companies interested in the genetic engineering of plants. Investigators at Hiram College in Ohio collaborated with a sequencing group at the Monsanto Company (Goodner et al., 2001) and the Crown Gall group and the genome sequencing team at the University of Washington in Seattle collaborated with scientists at E.I. DuPont de Nemours Company (Wood et al., 2001). Both sequences, which agreed with each other surprisingly well, were published simultaneously. These academic-industry collaborations were necessary because the genome was sequenced at a time when sequencing and annotating of even one relatively small bacterial genome required a major commitment of time, as well as significant intellectual and financial resources. The sequence revealed an extensive similarity of the circular chromosomes of *A. tumefaciens* and the plant symbiont, *Sinorhizobium meliloti*. This suggests that both organisms evolved from a common ancestor from which they recently diverged.

### CHARACTERIZATION OF DNA IN TUMORS

How does the Ti plasmid cause tumors? Because of the observations which strongly suggested that the TIP could be transferred between bacteria, the Seattle group searched for plasmid genes in tumor tissue. The technique they used is relatively simple in theory but very tedious in practice. Heat denatured ^32^P labeled DNA of the plasmid (probe) was mixed at a low concentration with a high concentration of unlabeled single-strand DNA isolated from either tumor or normal tissue (driver DNA). If plasmid genes were present in the tumor tissue, the concentration of these sequences would be elevated in the mixture. Consequently, the rate of reassociation of the single-strand probe DNA would be accelerated when mixed with tumor DNA as compared to the reassociation of probe DNA mixed with DNA from normal tissue. What was observed was a slight but reproducible increase in the rate of reassociation of the probe when a mixture of probe with tumor DNA was compared to probe mixed with normal plant DNA. These data could be explained if only a small fraction of the Ti plasmid was present in the tumor. This possibility was pursued through experiments designed to identify the putative piece of the Ti plasmid in tumor tissue (Chilton et al., 1977).

The plasmid was labeled with ^32^P then cleaved with a restriction enzyme. Sixteen fragments were separated by electrophoresis, then eluted from the gel and denatured. Each fragment was then mixed with normal and tumor DNA. This time the results were unequivocal. The reassociation of one doublet band mixed with tumor DNA showed a significant increase in the rate of reassociation. When this band was separated into two, one band showed no increase in reassociation whereas the other band increased the rate of reassociation even more than the doublet (Chilton et al., 1977). These data proved that the TIP of Braun was indeed DNA as others had previously suspected. This DNA was termed T-DNA for transferred DNA.

The identification of the TIP raised many interesting questions. Many laboratories studied the Ti plasmid. Some focused on the Ti plasmid from strains that induced tumors that synthesized octopine (octopine strains like A6 and B6). Others studied strains that induced nopaline synthesizing tumors, like C58 (nopaline strains). A few laboratories focused on *Agrobacterium rhizogenes*, a species of *Agrobacterium* that produces tumors, called hairy root because of a preponderance of roots at the site of inoculation (Moore et al., 1979; White and Nester, 1980). The virulence plasmid from this strain was termed the Ri plasmid for root inducing. The more common strains of *A. tumefaciens* like A6 and C58 result in unorganized tumor growth at the site of inoculation.

It was quickly determined that the T-DNA in tumors is transcribed, the first evidence that bacterial DNA can be transcribed in a eukaryotic cell (Drummond et al., 1977). Studying the patterns of transcription of the entire Ti plasmid revealed that the T-DNA was transcribed at a low rate in *Agrobacterium* whereas other regions of the Ti plasmid were transcribed to high levels (Gelvin et al., 1981). At least some of the T-DNA transcripts detected in the tumor originated and terminated within the T-DNA and not from promoters and terminators in the plant. The transcript levels of various regions of the T-DNA varied. All transcripts were polyadenylated (Gelvin et al., 1982; Willmitzer et al., 1982). Since the biological functions encoded by these genes were not yet known, the full significance of these data could not be appreciated. However, the use of promoters encoded in the T-DNA proved invaluable in the genetic engineering of plants.

Once a restriction map of an octopine Ti plasmid was generated (Chilton et al., 1978), studies on the Ti plasmid were aimed at identifying the various functions encoded on the plasmid. An early study was carried out by Holsters et al. (1980) on a nopaline plasmid and Ooms et al. (1981) on an octopine plasmid. They isolated insertion and deletion mutants using different transposons and mapped regions required for tumor formation including the T-DNA as well as another region distinct from the T-DNA. Some insertions in the T-DNA region resulted in tumors which did not synthesize an opine. They also demonstrated that regions required for tumor formation in the nopaline strain are homologous to regions required for tumor formation in an octopine strain.

Simultaneously and independently of these studies, the entire genome of an octopine strain was being mutagenized using a different transposon (Garfinkel and Nester, 1980). Mutants which mapped to the T-DNA gave rise to tumors with altered morphologies, either extensive root or shoot proliferation. These tumors still synthesized octopine but some insertions in the *T-DNA* did not. Other mutations distinct from the T-DNA region resulted in cells unable to catabolize octopine, proof that proteins required to synthesize octopine are not involved in its degradation. Interestingly, none of the single mutants in the T-DNA resulted in an avirulent strain. However, mutations in a region on the Ti plasmid distinct from the T-DNA did give rise to avirulent mutants. Further, about one-third of the mutations that affected tumorigenesis mapped to the chromosome. Thus, the genes associated with virulence are located at three different sites in the genome. Two sets map to the Ti plasmid. One set includes the T-DNA. The other includes genes we now know are required for the processing and transfer of the T-DNA, the *vir* genes. In addition, many genes in the circular chromosome, the *chv* genes, strongly affect virulence. The genes on the Ti plasmid function in various stages of plant cell transformation. Their major role is associated with plant cell transformation. However, most *chv* genes are pleiotropic. Their protein products are required for optimal plant cell transformation but they also play various roles in the physiology of the bacteria in the absence of the host plant.

#### Functional analysis of the T-DNA

To understand the mechanism by which *Agrobacterium* confers transformed properties on the plant requires an understanding of what the T-DNA encodes. To this end, Garfinkel et al. (1981) generated a fine-structure map of the T-DNA through site-directed mutagenesis in an octopine strain. Insertions in one region resulted in tumors that no longer synthesized octopine. Insertions in three other regions affected tumorigenesis. Insertions in one region resulted in tumors forming a massive amount of roots emanating from the tumor callus (*tmr* mutations). Insertions in another region resulted in tumors with shoots growing from the tumor callus (*tms* mutations). Insertions in another region resulted in unusually large tumors on certain plants (*tml* mutations). Insertions between each of these regions had no effect on tumorigenesis. Ooms et al. (1981) independently generated mutations in a similar Ti plasmid and made similar observations. In addition, they observed that supplying an auxin (naphthalene acetic acid) to developing tumors incited by a *tms* mutant or a cytokinin (kinetin) to a *tmr* mutant stimulated unorganized tumor formation on tomato plants. This suggested that the proliferation of shoots in the *tms* mutant and roots in the *tmr* mutant resulted from an imbalance of the two phytohormones.

The analysis of phytohormone levels in uninfected tobacco stem tissues, wild type tumors and tumors induced by *tmr* and *tms* mutants on tobacco stems supported this idea (Akiyoshi et al., 1983). Whereas the ratio of cytokinin (*trans*-ribosylzeatin) to auxin (indoleacetic acid) levels in wild-type tumors was 0.2, the same ratio was much lower in *tmr* tumors and much higher in *tms* mutants. A simple explanation was that the T-DNA encodes the enzymes of auxin and cytokinin synthesis, an interpretation shown to be correct when Akiyoshi et al. (1984) showed that the *tmr* gene encodes an enzyme of cytokinin synthesis, dimethylallyltransferase. Sequencing the region of the *tms* gene revealed that this region encodes two transcripts, both of which are involved in auxin synthesis (Klee et al., 1984). One locus, *tms1*, encodes a tryptophan mono-oxygenase and *tms2* encodes indole-3-acetamide hydrolase (Thomashow et al., 1984). Together, both enzymes convert tryptophan to the auxin indole-3-acetic acid (Thomashow et al., 1986).

#### Organization of T-DNA in tumors

Does the T-DNA replicate as a plasmid or is it integrated into the chromosome? If the latter, are there specific sites at which the T-DNA is integrated? Are the putative inserts stably integrated or can they jump to different locations? The studies of Thomashow et al. (1980) answered these questions. They defined the T-DNA in four tumor lines and reached the following conclusions: (1) the T-DNA is integrated into numerous sites in the plant DNA; (2) each line contains a “core” DNA which is co-linear with the Ti plasmid; (3) a given Ti plasmid does not always give rise to the same insertions; and (4) the number of insertions of “core” DNA varies in different tumor lines. We now know that the “core” DNA includes those genes responsible for the tumor phenotype.

#### Virulence (vir) region-overview

The one region on the Ti plasmid in which mutations result in a complete loss of virulence is the *vir* region. It comprises ∼30 genes of which about 20 are essential for tumor formation. They are organized into operons which together comprise a regulon under a common control mechanism (Stachel and Nester, 1986; Gelvin, 2003). To study the genetic and transcriptional organization of this region, Stachel et al. (1985a) developed a Tn3-*lacZ* transposon and generated a random series of mutations. After analyzing 124 insertions for tumor formation and beta-galactosidase in bacteria grown in the presence of plant cells, they divided the *vir* region into six complementation groups: *virA*, *virB*, *virC*, *virD*, *virE*, and *virG* (Stachel and Nester, 1986). Mutations in *virA*, *B*, *D*, and *G* resulted in a complete loss of virulence. Mutations in *virC* and *virE* led to attenuation. Another *vir* gene, *virF*, is required for robust tumor formation on some, but not on other plants (Melchers et al., 1990). Another locus, *virH* (*pin*), is not required for virulence on any plant (Stachel and Nester, 1986). Additional *vir* genes that are not required for tumor formation on several plants are *virK*, *virL*, and *virM* (Kalogeraki and Winans, 1998) and a gene involved in cytokinin synthesis (*tzs*) found only in nopaline strains (Holsters et al., 1980). To observe any beta-galactosidase activity from the *lacZ* gene, bacteria had to be co-cultivated with plant cells because the expression of this region is under the tight control of plant metabolites (Stachel et al., 1986a).

Three signal molecules, all associated with the wound site on a plant are important in *vir* gene induction. These include a number of different phenolic compounds, a variety of monosaccharides which are components of plant cell walls and act through a binding protein encoded on the bacterial chromosome (ChvE) and acidic conditions which are required at several steps in the induction process. A two-component regulatory system is critical for recognizing all three signal molecules.

## MECHANISM OF ACTIVATION OF *vir* GENES-TWO-COMPONENT SYSTEM

Once the *vir* regulon was defined genetically, studies were aimed at understanding how these critical genes are regulated by the three types of signal molecules. The first clue came when it was found that mutations in either *virA* or *virG* eliminated induction of all *vir* genes (Stachel and Zambryski, 1986; Winans et al., 1988). Insight into how these two genes functioned came through gene sequencing and comparing the sequences with other regulatory gene pairs. Simultaneously with these studies on *Agrobacterium*, Fred Ausubel at Harvard was sequencing the nitrogen assimilation regulatory genes, *ntrB* and *ntrC* of *Bradyrhizobium*. After comparing the sequences of these two systems, both groups concluded independently that many regulatory systems that respond to environmental stimuli share strongly conserved domains (Nixon et al., 1986; Winans et al., 1986; Ronson et al., 1987).

How *VirA* and *VirG* function in regulating the *vir* genes was helped enormously by data generated in two similar systems in other bacteria, NtrB (nitrogen metabolism) and CheA (chemotaxis; Nixon et al., 1986). Taking cues from these other systems, VirA can be designated as the sensor protein which recognizes the plant signal molecules and VirG, the response regulator, which activates all genes in the regulon. As in these other systems, it was shown that VirA is an autophosphorylase which phosphorylates a specific histidine moiety (Jin et al., 1990a) and then transfers the phosphate to a specific aspartic acid in VirG, thereby activating the molecule (Jin et al., 1990b). The activated VirG then binds to a conserved 12 base pair sequence upstream of each of the *vir* genes (Das et al., 1986; Jin et al., 1990c).

### VirA

Insight into how the sensor protein VirA functions was provided by its sequence (Leroux et al., 1987). The deduced protein has two hydrophobic regions which suggests it is imbedded in a membrane. Using antibodies, the protein was shown to be anchored in the inner membrane with ∼275 amino acids near the amino terminus localized in the periplasmic space and the rest of the protein located in the cytoplasm. Other members of the family of homologous proteins have a similar hydropathy profile (Charles et al., 1992). VirA exists as a preformed dimer in the cell (Pan et al., 1993).

Later genetic studies divided the VirA protein into several additional domains which function independently of one another and to which functions were assigned (Chang and Winans, 1992). The periplasmic domain is required for the sensing of monosaccharides (Cangelosi et al., 1990a). A linker domain joins the transmembrane region to the cytoplasmic region which includes the kinase and receiver domains. The linker domain is required for the sensing of phenolic compounds and acidity whereas the kinase domain contains the phosphorylatable histidine moiety. The receiver domain serves as an enhancing region of VirA and is required for *vir* gene expression (Wise et al., 2010).

### VirG

The *virG* locus is transcriptionally activated by plant signal molecules acting on one of two promoters, P1 and P2 located downstream of P1 (Mantis and Winans, 1992). The P1 promoter functions with phenolic inducers and phosphate starvation and requires the VirA/G system. In contrast, the P2 promoter is activated solely by acidic conditions which serves to raise the level of VirG to the level required to achieve maximum induction of the *vir* regulon by phenolic and monosaccharide inducers. Acid induction is independent of the VirA/G system (Mantis and Winans, 1992).

## PLANT SIGNAL MOLECULES

That it was necessary to co-cultivate *Agrobacterium* with plant cells in order to observe expression of the *vir* genes, suggested that plant cells were secreting molecules that induced the *vir* regulon (Stachel et al., 1986a). The identification and functional characterization of the three inducing molecules represents one of the first examples of our understanding, albeit incomplete, of how a bacterial cell responds to its complex, natural environment.

### PHENOLIC INDUCERS

Two low molecular weight phenolic compounds, dimethoxyphenol [acetosyringone (AS) and hydroxyacetosyringone (OH-AS)], secreted by tobacco cells and at biologically relevant amounts were shown to induce the *vir* genes (Stachel et al., 1985b). This was a key discovery in crown gall research and made many other investigations possible. A whole host of naturally occurring phenolic plant metabolites were later shown to be inducers (Brencic et al., 2004). These included vanillin, coniferyl alcohol, sinapyl alcohol, syringaldehyde, and eugenol.

Although the model of phenolic signaling strongly suggests that the phenolic molecule must bind, directly or indirectly, to VirA, such binding has never been demonstrated biochemically. However, genetic evidence is consistent with this model (Lee et al., 1995). By transferring different Ti plasmids having different specificities for *vir* gene inducing phenolic compounds into isogenic chromosomal backgrounds, it was shown that the specificity of *vir* gene activation by these different phenolic compounds tracks with the *virA* locus.

### MONOSACCHARIDES-NEUTRAL AND ACIDIC

A surprisingly wide range of monosaccharide components of plant cell walls act in concert with phenolic inducers to increase the level of induction achieved by phenolic compounds alone. Many different neutral and acidic sugars are recognized by a chromosomally encoded protein, ChvE (Ankenbauer and Nester, 1990; Cangelosi et al., 1990a; Shimoda et al., 1990; He et al., 2009; Hu et al., 2013). Once bound to a sugar, ChvE can bind to the periplasmic region of VirA (Cangelosi et al., 1990a; Shimoda et al., 1993), relieve repression and transduce this information through the cytoplasmic membrane domain to activate the kinase domain which then transfers its phosphate to VirG (Nair et al., 2011). Mutations in *chvE* resulted in the *vir* genes being poorly inducible both in the maximum level of induction achieved and also in their level of induction at low concentrations of the phenolic inducer. Also, such mutants were less virulent (Cangelosi et al., 1990a; Shimoda et al., 1990; Nair et al., 2011). ChvE mutants were also defective in chemotaxis and grew poorly on a variety of sugars which are involved in *vir* gene induction (Cangelosi et al., 1990a; He et al., 2009). Sequencing the gene which encodes the ChvE protein revealed that it is homologous to the glucose/galactose binding protein of *E. coli*, which plays a role in the uptake of sugars as well as chemotaxis. Mutants defective in ChvE have an altered host range. They remain virulent on some plants but avirulent or weakly virulent on others. These differences are likely a consequence of the level of *vir* gene induction required for plant infection (Banta et al., 1994; Nair et al., 2011). Some plants are more susceptible to infection than others and the degree of susceptibility is reflected in the level of *vir* gene products required for a successful infection (Cangelosi et al., 1990a). The *chvE* locus represents an excellent example of the pleiotropic nature of a chromosomal gene which is important in virulence but also plays a role in the physiology of the organism growing in the absence of the plant. This pleiotropic nature of ChvE will be covered in more detail shortly.

### ACIDIC CONDITIONS

Acidic conditions (pH 5.5) play a critical role in *vir* gene induction through a number of different mechanisms some of which are not well understood or even recognized. One important function is to raise the level of the response regulator, VirG, to the level necessary for maximum *vir* gene induction (Mantis and Winans, 1992). This occurs in the absence of the phenolic and sugar inducers. Apparently, the elevated level of VirG then becomes sufficient to induce all genes of the *vir* regulon, including *virA* and *virG*, through signal molecules at the wounded plant site. Acidic conditions are also required for the binding of sugars to ChvE (Hu et al., 2013).

To expand the repertoire of genes affected by an acidic environment, a transcriptomic analysis of cells grown at pH 5.5 and pH 7.0 was carried out (Yuan et al., 2008). Seventy eight genes were significantly induced and 74 repressed at pH 5.5. Of the genes induced, 17 were involved in the synthesis of the cell envelope. This may reflect the need of an altered cell surface to associate with the plant cell. Of special interest was another two-component regulatory system that was strongly induced, ChvG/I. This system was identified previously by screening avirulent mutants that mapped to the chromosome (Charles and Nester, 1993). It was isolated independently by another screen (Mantis and Winans, 1993). This complex regulatory system will be discussed later when chromosomal mutants associated with virulence are considered.

Another set of genes that was strongly induced were the genes involved with a Type VI secretion system (Yuan et al., 2008; Wu et al., 2012). This system mediates interaction between a wide variety of bacteria by acting as an export channel for the transfer of various kinds of toxins into neighboring cells following cell–cell contact (Russell et al., 2014). The significance of this system to the association of *Agrobacterium* and plants is not clear. However, work in *Agrobacterium* and other systems suggests that this secretion system may provide a mechanism for *Agrobacterium* to achieve a competitive advantage with other organisms in the acidic environment of the rhizosphere (Ma et al., 2014; Russell et al., 2014).

## Vir PROTEINS

As discussed already, two of these proteins VirA and VirG are concerned with the activation of all *vir* genes. The other Vir proteins are required for the processing and transfer of the T-DNA.

### VirB

Perhaps the most intriguing operon of the *vir* regulon is *virB*, in part because of its large size. The entire operon was sequenced and 11 open reading frames identified (virB1-11; Ward et al., 1988). Many encode presumed gene products with secretion signals and membrane spanning domains. The sequence analysis suggested that this operon encodes a transmembrane structure that likely mediates the passage of the T-DNA and certain Vir proteins into the plant cell. VirB is now the paradigm for the intensely studied Type IV secretion system found in a wide variety of prokaryotic cells. More is known about the structure-function relationships of the Type IV secretion apparatus in *Agrobacterium* than in any other organism (Cascales and Christie, 2004; Alvarez-Martinez and Christie, 2009).

Recently, the structure of another well-studied Type IV secretion system in *E. coli* has been elucidated in great detail (Low et al., 2014). The secretion system from the conjugative plasmid R388 was over-expressed in *E. coli*. The structure consists of a core complex which is joined to the inner membrane complex by a stalk. Many but not all of the constituent proteins were localized in the structure. VirB1 which was not included encodes a transglycosylase which cleaves beta-1,4 glycosidic bonds (Mushegian et al., 1996). VirB2 encodes the synthesis of pilin, the subunit of the T-pilus (Lai and Kado, 1998). The synthesis of T-pilin is temperature sensitive (Fullner et al., 1996; Lai and Kado, 1998). Interestingly, the intact T-pilus is not required for transfer of T-DNA but its subunit is (Kerr and Christie, 2010).

### VirD

The *virD* operon consists of five open reading frames. Interestingly, the encoded proteins play quite different roles in the infection process. VirD2 is an endonuclease that nicks one of the two strands of the Ti plasmid at two sites which flank and delineate the T-DNA (Yadav et al., 1982; Yanofsky et al., 1986). These 25 base pairs occur as direct repeats and their cleavage results in the formation of a single-strand T-DNA molecule, the T-DNA (Stachel et al., 1986b; Albright et al., 1987; Veluthambi et al., 1988). The VirD2 protein remains covalently attached at the 5^′^ end through a phosphotyrosine bond (Ward and Barnes, 1988; Young and Nester, 1988; Pansegrau et al., 1993). This protects the 5^′^ end from exonucleolytic degradation (Durrenberger et al., 1989). In addition to the indispensable border sequences, efficient T-DNA transmission requires an additional sequence, termed overdrive which is located to the right of the right border (Peralta et al., 1986) and serves to enhance the production of T-strands (Veluthambi et al., 1988).

Following nicking, the VirD2 protein which also contains nuclear localization signals (NLSs) directs the transport of the T-DNA into the nucleus of the plant cell (Herrera-Estrella et al., 1990; Howard et al., 1992). In addition, this protein is in part responsible for the efficiency of transformation and the preservation of the ends of the integrated DNA (Tinland et al., 1995; Pelczar et al., 2004).

The VirD2 protein also carries the translocation signal for T-strand docking with the VirD4 coupling protein. This latter protein has ATPase activity and delivers the T-strand to the mating pair channel, the *trans*-envelope secretion system encoded by the *virB* operon. The binding of the T-DNA to VirD4 and VirB11, which also has ATPase activity, stimulates their ATPase activities. This energy in turn activates VirB10 through a structural transition which opens up the channel to the cell surface. The ATPase activities of VirD4 and VirB11 as well as the binding of the T-DNA to VirD4 are required to activate VirB10 through a structural modification (Cascales et al., 2013).

### VirC

The virC operon consists of two open reading frames, *virC1* and *virC2* (Stachel and Nester, 1986). Mutations in either result in attenuated virulence on some but not all plants (Yanofsky et al., 1985). Presumably the plants on which the mutations do not effect virulence are those most susceptible to infection. This operon appears to function at several early stages in the transformation process. VirC1 binds to the overdrive sequence and enhances the site-specific nicking by the VirD endonuclease thereby resulting in increased T-strand production (Toro et al., 1988, 1989). Although the *virC* operon is not essential for endonuclease nicking, VirC1 does enhance nicking, most likely because of the interaction between VirC1 and overdrive. More recent studies demonstrated that VirC2 increases the number of copies of T-strands per cell as a result of the pair-wise interactions with VirD2, VirC1, and VirD1 which most likely exist as multimers (Atmakuri et al., 2007). In addition to its role in T-strand formation, VirC1 recruits the cytosolic T-strands to the type IV secretion channel.

### VirE

The *virE* operon consists of three open reading frames, of which the most intensely studied is *virE2*. *virE2* encodes a non-specific single-strand DNA binding protein which likely covers the length of the T-DNA (Gietl et al., 1987; Christie et al., 1988; Citovsky et al., 1988; Das, 1988; Yusibov et al., 1994). It is delivered via the Type IV secretion system into the plant cell independent of T-DNA transfer where it presumably protects the T-DNA against nuclease degradation and maintains the integrity of the 3^′^ end of the T-DNA prior to integration (Rossi et al., 1996). Unlike *virE2* and *virE1*, *virE3* encodes a host range locus and will be considered in a later section.

Although VirE2 also contains NLS, data conflict as to whether these signals play a significant role in the nuclear import of T-DNA. Early studies strongly suggested that the NLS of VirE2 were very important in nuclear import. These studies indicated that they were important both in localizing the T-DNA into the nucleus (Zupan et al., 1996) and also in virulence (Gelvin, 1998). In the latter study, a VirE2 mutant lacking NLS was avirulent but regained virulence on tobacco plants that expressed VirE2. Another report distinguished between nuclear targeting and nuclear import (Ziemienowicz et al., 2001). These authors concluded that VirD2 was necessary to target the DNA to the nucleus but VirE2 was necessary for its import. A recent study however did not show that VirE2 localized to the nuclei of yeast and tobacco cells (Sakalis et al., 2014). Using several different visualization techniques, these investigators demonstrated that VirE2 traveled from *Agrobacterium* into plant cells where it associated with microtubuli. However, the interaction with the microtubules might be merely one step on the way to the nucleus. This is only the latest study of many which did not show localization of VirE2 in the nucleus (Reviewed in Gelvin, 2012; Lacroix and Citovsky, 2013). Some of the conflicting localization data reported might be resolved by results reported by Bhattacharjee et al. (2008). They observed that VirE2 localized to the cytoplasm of *Arabidopsis* cells but that over-expression of a specific isoform of importin, IMPa-4, a nuclear transport factor which interacts with VirE2, resulted in the VirE2 now localizing in the nucleus. This suggests that the level of this specific isoform could determine whether VirE2 localizes to the cytoplasm or the nucleus. Since different plants and different parts of plants would likely have different levels of this importin, this could explain conflicting results from different studies. Further, reducing the level of IMPa-4 reduced the level of plant cell transformation, also suggesting that this specific importin, and by implication VirE2, is important in nuclear transport.

#### VirE1

Another gene in this operon is *virE1*. This gene encodes a chaperone which keeps the VirE2 protein from aggregating with itself inside *Agrobacterium* (Sundberg et al., 1996; Deng et al., 1999). It cements two independent domains of VirE2 into a locked form, thereby creating a soluble heterodimer (Dym et al., 2008). Further, VirE1 competes with the T-strand for binding to VirE2 inside the bacterial cell but unlike VirE2, VirE1 apparently does not enter the plant cell (Dym et al., 2008).

### NON-ESSENTIAL AND HOST RANGE Vir PROTEINS

The mutational and sequence analysis of the *vir* regulon revealed many open reading frames whose products seemed to be non-essential for tumor formation on the plants tested (Kalogeraki and Winans, 1998; Kalogeraki et al., 2000). In some cases, however, assays on additional plants showed that they were important for tumor formation. However, some loci still seem to play no role in tumor formation on any plants at least in laboratory assays.

#### VirH

The *virH* region consists of two genes which encode proteins which resemble P-450-type monooxygenases (Kanemoto et al., 1989). Such genes are usually associated with detoxification of a variety of compounds. A strain mutant for both genes was still tumorigenic on *Kalanchoe daigremontiana* and carrot disks (Kalogeraki and Winans, 1998). Each gene encodes a protein that can convert a strong phenolic *vir* gene inducer, ferulic acid, to a non-inducer, caffeate by demethylating a methoxyl group. Most *vir* gene inducers are toxic to *Agrobacterium* and in general their conversion to non-inducers relieves this toxicity. However, not all *vir* gene inducers are demethylated at an appreciable rate so it is unclear what role these proteins play in the plant environment (Brencic et al., 2004).

#### VirF

Mutants lacking this gene in an octopine strain formed normal appearing tumors on *Nicotiana tabacum* and *Kalanchoe.* However, the same mutants were highly attenuated on *Nicotiana glauca* and tomato (Regensburg-Tuink and Hooykaas, 1993). Importantly, transgenic plants expressing the *virF* gene became transformable by these same mutants, thus indicating that VirF functions in the plant cell. Vir F is an F-box protein, representatives of which are part of the SCF complex which mediates ubiquitination of proteins targeted for degradation by the proteasome. This suggests that VirF may be involved in proteolysis of proteins such as VirE2. Any protein associated with the T-DNA presumably must be stripped prior to DNA integration. In support of this idea, VirF can mediate targeted proteolysis of VirE2 both in yeast and *in planta* (Tzfira et al., 2004). Interestingly, VirF has a functional ortholog (VBF) in some plants whose synthesis is induced by *Agrobacterium* infection. This ortholog can supply VirF function in *Agrobacterium* strains lacking VirF (Zaltsman et al., 2010). Reducing the level of this ortholog in plants increases their resistance to *Agrobacterium* infection.

#### VirD5

Another exported protein which originally was underappreciated is VirD5. The VirF protein is unstable in plants apparently because of degradation by the plant’s ubiquitin proteasome system (Magori and Citovsky, 2011). To overcome this instability, *Agrobacterium* transfers VirD5 into the plant where it binds to VirF and prevents its rapid turnover.

#### VirE3

VirE3 is presumably another host range virulence factor. Like VirF, VirE3 has a functional ortholog in plants, VIP1. This transcription factor with NLS was reported to be important in plant cell transformation because it interacts with VirE2 and functions in the nuclear import of T-DNA (Tzfira et al., 2001). However, the importance of VIP1 in plant cell transformation has been brought into question by a recent study which presents convincing evidence that VIP1 is not required for transformation of at least several plants (Shi et al., 2014). Therefore, VIP1 and presumably its ortholog VirE3 must function in some capacity other than helping VirE2 enter the nucleus. Perhaps relevant to this conundrum is a study which reported that VirE2 does not interact with importin IMPa and therefore must require another factor to interact with other than VirE2 for nuclear import (Ballas and Citovsky, 1997). Presumably this is VIP1. However, a more recent study showed that VirE2 does indeed associate with this specific importin. Therefore there is no need to postulate that another plant factor must be involved in nuclear import of VirE2 (Bhattacharjee et al., 2008). The significance of studies which reported that VirE3 can complement a VIP1 mutation both in nuclear import of T-DNA as well as susceptibility to transformation is unclear (Lacroix et al., 2005). Meanwhile, the function(s) of VIP1 and VirE3 remains unresolved.

#### VirJ

The VirJ protein is encoded by the *virJ* gene which maps between *virA* and *virB* and is the only copy present in nopaline strains (Pan et al., 1995). Mutants lacking this gene are avirulent (Wirawan et al., 1993). Octopine strains contain a functional chromosomal copy of *virJ*, labeled *acvB* which is constitutively expressed as well as the copy on the Ti plasmid (Pan et al., 1995). Agroinfection studies indicated that these mutants cannot transfer T-DNA into host cells (Pan et al., 1995). However, the exact role of this protein in T-DNA transfer is unclear. The protein is located in the periplasm and by immunoprecipitation assays bound to all VirB proteins (Pantoja et al., 2002). However, neither VirJ nor AcvB interact with the T-strand and do not appear to be subunits of the Type IV secretion system (Cascales and Christie, 2004). Its role in T-DNA transfer remains an intriguing mystery.

#### Small heat shock protein (HspL)

A proteomics analysis of AS induced genes in *A. tumefaciens C58* (a nopaline strain) revealed two new Vir proteins (Lai et al., 2006). One, Y4mC, which maps in the *vir* region, is not found in octopine strains. Whether it plays any role in virulence is unknown. The other is a small heat shock protein which is chromosomally encoded. Although it requires the VirA/G two-component system for its synthesis, it does not have a canonical vir box in its promoter region. This suggests that its synthesis depends on the expression of other genes under the control of VirA/G. This specific heat shock protein is located in the membrane where it functions as a chaperone for the VirB8 protein, a component of the structure of the Type IV secretion system (Tsai et al., 2012).

## CHROMOSOMAL vir PROTEINS (Chv)

Mutations in the chromosome as well as in the Ti plasmid can lead to alterations in virulence. The *chv* genes are not induced by AS, although many are induced by acidic conditions (Yuan et al., 2008). Analyzing many of these mutations has led to the conclusion that most of these genes encode proteins that play important roles in the physiology of the organism growing independently of the plant cell environment. However, these proteins directly or indirectly also play important roles in the interaction of *Agrobacterium* with plants. The pleiotropic nature of these functions make their exact role in tumor formation generally difficult to unravel. For purposes of discussion, *chv* mutations can be divided into several different categories, recognizing that these are subject to change as additional information is gained about them. These categories are: (1) mutations affecting expression of *vir* genes, (2) mutations involved in membrane structure, and (3) mutations involved in plant response.

### MUTATIONS AFFECTING EXPRESSION OF *vir* GENES

#### ChvE

The *chv* gene encodes a periplasmic binding protein and probably represents the most intensely studied of all the Chv proteins. Recent studies show just how important this protein is in plant cell transformation. Its role in signaling in the VirA/G system has already been discussed. However, it also binds to sugar transport as well as chemotaxis proteins (Cangelosi et al., 1990a; Kemner et al., 1997; Zhao and Binns, 2011). In these multiple functions, the N-terminal and C-terminal domains of ChvE that interact with VirA partially overlap the surface required for binding to the sugar transport protein (He et al., 2009) and thus the sugar uptake system competes with VirA as a receptor for ChvE. This competition has important consequences in determining the strength of phenolic compounds to serve as inducers of the *vir* genes (Hu et al., 2013). Thus, a specific inducing sugar can increase the inducing capability of a weak phenolic inducer if the level of ChvE is increased. Consequently, the ability of various phenolic compounds to serve as inducers depends qualitatively and quantitatively on the level of ChvE interacting with VirA (Peng et al., 1998).

Mutating the *chvE* locus at several different sites resulted in mutant ChvE proteins which responded differently to different neutral and acidic sugars in *vir* gene expression (Hu et al., 2013). Testing these mutants for their virulence on several different plants demonstrated that different sugars limit tumor formation depending on the host plant. Also, in at least one common and well studied strain of *Agrobacterium*, C58, ChvE is essential for *vir* gene induction even in the presence of high levels of AS. (Doty et al., 1996) and ChvE is required for successful agroinfection of maize (Raineri et al., 1993). Thus, ChvE can be considered a host range determinant whose biological importance in the physiology and tumor-inducing capabilities of *Agrobacterium* still remains incomplete.

#### ChvG/I

An intriguing *chv* region encodes the two-component system, ChvG/I. Mutants were isolated and characterized independently in two laboratories and were shown to be avirulent (Charles and Nester, 1993; Mantis and Winans, 1993). Mutants in either the sensor protein (ChvI) or response regulator (ChvG) are pleiotropic resulting in cells which are weakly inducible, are unable to grow on a rich medium, are sensitive to detergents, wound sap and acidic conditions. Further, the *virG* locus is no longer induced by acid (Charles and Nester, 1993; Mantis and Winans, 1993) although acid induces the expression of *chvG/I* (Yuan et al., 2008). Elevating the level of VirG by placing this locus under the control of an inducible *lac* promoter in a *chvI* mutant did not rescue *vir* gene expression (Mantis and Winans, 1993) suggesting that one effect of the *chvI* mutation must be downstream of the expression of *virG*.

The ChvG/I system is a global regulator of many acid-inducible genes many of which are required for virulence (Li et al., 2002). These include genes on the Ti plasmid and the two chromosomes and include the acid induction of *virG*. This is consistent with data that a chromosomal gene is responsible for the acid induction of *virG* (Mantis and Winans, 1992). This system in turn is under the control of ExoR, a periplasmic, acid sensitive protein which apparently binds to ChvG and prevents phosphate transfer to ChvI (Wu et al., 2012; Heckel et al., 2014). However, under acidic conditions, ExoR is subject to specific proteolysis which allows ChvI to be activated which promotes binding to upstream regions of acid-inducible genes. This model, in part, is based on data from a homologous system in *Sinorhizobium* (Chen et al., 2008, 2009; Lu et al., 2012).

#### ChvH

A mutation in the *chvH* locus results in pleiotropic mutants that are highly attenuated in virulence, synthesize much lower levels of VirG and VirE as well as VirB8, VirB9, VirB10, and VirB11 (Peng et al., 2001). Further, the mutants are highly sensitive to detergents and carbenicillin, and grow more slowly than the parental strain. In contrast to the decrease in the levels of Vir proteins which were synthesized, a 32 kDa protein accumulated which was not identified. Thus, the ChvH protein appears to selectively alter the level of certain proteins in the cell and specifically those that contribute to certain stress responses, such as acidic conditions, sensitivity to detergents and penicillin. DNA sequencing revealed that the *chvH* locus encodes an elongation factor P (EF-P), a translation factor that stimulates the formation of the first peptide bond in protein synthesis (Blaha et al., 2009). The mutation is highly conserved since the mutation can be complemented by the homologous *ef-p* locus from distantly related *E. coli* (Peng et al., 2001).

Recent studies on the EF-P protein in *E. coli* and *Salmonella* revealed that this post-transcriptional regulatory pathway is also essential for virulence of *Salmonella* and the synthesis of functional membranes, a story similar to what was observed in *Agrobacterium* (Zou et al., 2011, 2012). Thus, the *chvH* locus encodes a novel control system that regulates a limited number of proteins some of which are important in virulence and the stress response of *Agrobacterium*. What is the mechanism for this regulation? The protein EF-P interacts with the ribosome and specifically facilitates the synthesis of proteins with the specific amino acid motifs PPP and PPG (Hersch et al., 2013). The loss of EF-P would result in a limited synthesis of such proteins, apparently a feature of many of the Vir proteins. The factors that regulate ChvH synthesis are not known but do not appear to include acidic conditions (Yuan et al., 2008).

#### ChvD

This mutant protein was identified in a screen for mutants with altered expression of *virG*. The chromosomal mutant demonstrated reduced acid induction of *virG* gene expression and was also attenuated in virulence (Winans et al., 1988). Gene sequencing revealed that the predicted protein sequences were homologous to a family of proteins involved in active transport (Winans et al., 1988). Introduction of a constitutive *virG* locus into the *chvD* mutant restored virulence, indicating that the effect of the mutation on virulence is associated with its effect on *virG* expression (Liu et al., 2001). However, how a mutation in this putative transport system affects *virG* expression is not at all clear.

#### Citrate synthase

This mutant, blocked in the synthesis of citrate in the tricarboxylic acid (TCA) cycle was isolated as a Tn*PhoA* insertion mutant that is attenuated in virulence (Suksomtip et al., 2005). The mutation resulted in a 10-fold reduction in *vir* gene expression. Introduction of a constitutive *virG* locus into the mutant restored both *vir* gene expression and virulence. Thus the mutant defect must be upstream of the VirA/G regulatory system but the basis of this effect is not known. The addition of several intermediates of the TCA cycle had no effect on *vir* gene expression. Surprisingly, the mutant cells grew almost as well as the wild type cells on minimal medium and restoring *vir* gene induction had no effect on the growth rate. Thus the reduced growth rate cannot account for the attenuation of virulence of the mutant.

#### Phosphoenolpyruvate carboxykinase (PckA)

The *pckA* gene encodes a protein which catalyzes the reversible decarboxylation and phosphorylation of oxaloaceate to form phosphoenolpyruvate. The locus maps to the circular chromosome and interestingly is located immediately downstream of the *chvG/I* genes but is transcribed in the opposite direction. This gene is acid inducible, and like other acid inducible genes is under the control of ChvG/I. *pckA* mutants grew more slowly under acidic conditions than did the parent cells (Liu et al., 2005). They were highly attenuated in virulence and also in *vir* gene expression. However, introduction of a constitutive *virG* locus into the mutant restored *vir* gene induction to wild-type levels but only partially restored virulence. Neither were the growth rates restored at pH 5.5 or pH 7.0. The continued sensitivity of the mutant to growth inhibition under acidic conditions may account for the lack of complete restoration of virulence in the mutant with a constitutive *virG* gene. Why a mutation in the *pckA* gene should reduce *vir* gene expression and why its expression is acid inducible remain a mystery.

### MUTATIONS INVOLVED IN MEMBRANE STRUCTURE

#### ChvA and ChvB

Other *chv* mutants with pleiotropic effects are those unable to synthesize or transport beta-1,2-glucan molecules into the periplasm. They are designated *chvB* and *chvA* respectively. They were isolated as mutants unable to attach to plant cells and therefore are avirulent (Douglas et al., 1982, 1985). On low osmotic strength media, the mutants grew more slowly than wild-type cells and exhibited an altered periplasmic and cytoplasmic protein content as well as reduced motility. When returned to a high osmotic strength medium, their growth rate and protein content were restored to wild-type levels. However, the mutants were still avirulent and had reduced motility regardless of the media (Cangelosi et al., 1990b). These data suggest that the periplasmic glucan plays a role in osmoadaptation although its role in virulence and motility may be only indirectly related to its role in osmoadaptation. Addition of beta-1,2-glucan to both mutants did not affect their ability to bind to plant cells. It seems highly unlikely that the glucan is directly involved in binding. That *Agrobacterium* can attach to so many different hosts suggests that binding may not be very specific.

#### Agrobacterium outer membrane protein (AopB)

AopB is an outer membrane protein in which mutations lead to a highly attenuated virulence phenotype (Jia et al., 2002). The encoding locus maps to the circular chromosome, is acid inducible and shares high homology with a gene from *Rhizobium leguminosarum*. Like other acid inducible loci, this gene is also under the control of ChvG/I (Yuan et al., 2008). The *vir* genes are expressed normally. There is some sequence similarity to genes encoding porin proteins suggesting that this protein may be involved in transport functions, like ChvD, but its role in tumorigenesis is unknown.

### MUTATIONS INVOLVED IN PLANT RESPONSE

#### tRNA: isopentenyltransferase (MiaA)

This Tn5 induced mutation involves the locus that is responsible for the specific modification of a specific residue in a codon of a tRNA species (*miaA* gene). The mutation reduced *vir* gene expression 2- to 10-fold when induced by AS (Gray et al., 1992). Virulence was reduced on some but not on other plants. Sequence analysis revealed an open reading frame with a strong homology to the *miaA* gene from *E. coli*.

The understanding of how a mutation in the *miaA* gene reduces tumorigenesis in some plants took more than 20 years to solve. In addition to the *miaA* locus, the story involves the *tzs* gene of the *vir* regulon found in nopaline but not octopine strains and a plant product, MTF, a transcription factor. The gene products of both *tzs* and *miaA* involve cytokinin synthesis, a phytohormone which has been implicated in promoting tumorigenesis (Zhan et al., 1990; Chateau et al., 2000; Hwang et al., 2010). Mutations in the *miaA* gene or loss of the *tzs* locus result in reduced levels of secreted cytokinin (Regier and Morris, 1982; Gray et al., 1996). Recently, the molecular basis of cytokinin action on the plant has been elucidated. Cytokinin decreases the expression of a plant transcription factor, MTF, which normally inhibits plant cell transformation (Sardesai et al., 2013). Thus, any mutation that reduces the level of secreted cytokinin decreases the susceptibility of the plant to transformation by *Agrobacterium*. Apparently, the MTF transcription factor may control the synthesis of plant receptors to which *Agrobacterium* may attach. As predicted, mutating MTF increases plant cell transformation (Sardesai et al., 2013). However, these impressive studies do not explain why *vir* gene expression is reduced in a MiaA mutant. Does cytokinin modulate *vir* gene expression?

#### Catalase (KatA)

A transposon induced mutation screen for acid inducible genes identified a gene (*katA*) encoding a catalase (Xu and Pan, 2000). This enzyme catalyzes the dismutation of hydrogen peroxide to water and oxygen and its synthesis is acid inducible (Li et al., 2002). Hydrogen peroxide must provide an important defense to the *Kalanchoe* plant because mutants lacking catalase are weakly virulent. This is the only *chv* gene thus far identified whose gene product apparently serves a single function.

## HOST RANGE

*Agrobacterium* has a remarkably broad host range especially considering other plant pathogens. Initially, plant infection was assayed by tumor formation, a measure of both T-DNA transfer and integration into the plant chromosome. Using this assay, a very large number of monocots and dicots were tested with the conclusion that although most dicots were susceptible to varying degrees, no monocots were (DeCleene and DeLey, 1976). This conclusion was shown to be an overgeneralization when it was demonstrated that *Agrobacterium* could form tumors on the monocot, *Asparagus* (Bytebier et al., 1987). Also, it was clear that the host range of *Agrobacterium* could be expanded to include certain poorly transformable plants by increasing the level of expression of the *vir* genes (Jin et al., 1987; Gelvin, 2003). “Super virulent” strains were constructed that proved useful in infecting many recalcitrant dicots (Hood et al., 1986; Jin et al., 1987; Reviewed in Banta and Montenegro, 2008). It was also clear that the defense a plant mounts against the invading *Agrobacterium* plays an important role is determining whether an infection is successful (Reviewed in Pitzschke, 2013). However, for whatever reason, all attempts to show tumor formation on cereal plants proved negative. Some plant scientists in the mid 1980s believed that the biology of monocots and dicots might be so different that monocots were inherently non-transformable.

A number of laboratories continued their attempts to understand why monocots were resistant to infection by *Agrobacterium* using several different assays. It was clear that tumor formation was a poor assay since monocots do not respond to increased levels of phytohormones as do dicots and therefore gene transfer might occur without gall formation. Therefore genes encoding color markers such as beta-glucuronidase and antibiotic resistance were substituted as markers for T-DNA transfer, but not necessarily integration. These assays were fast, and could be carried out in Petri dishes testing a variety of bacterial strains, plant tissues, and environmental conditions.

An especially clever and elegant assay allowed Barbara Hohn and her colleagues to demonstrate that *Agrobacterium* can transfer T-DNA into maize plants (Grimsley et al., 1987). In this assay, a viral genome, maize steak virus, was inserted into the T-DNA and maize plants were inoculated with *Agrobacterium* containing the viral genome (Grimsley et al., 1986). The appearance of viral symptoms at the site of inoculation indicates that the T-DNA has been transferred, the viral genome excised from the T-DNA and the virus has replicated. This assay, termed agroinfection, is a very sensitive measure of gene transfer because virus replication magnifies a single T-DNA transfer event. Thus, it was possible to readily study the features of *Agrobacterium* important for successful gene transfer independent of integration. Using this assay, it was shown that only certain strains of *Agrobacterium* could agroinfect. Thus, octopine strains could not, but nopaline strains could (Boulton et al., 1989). Introducing the *virA* gene from the nopaline into the octopine strain converted the latter strain into an agroinfected (Raineri et al., 1993). Dissecting the VirA protein into distinct regions revealed that the linker region of VirA is especially important for successful agroinfection. Further, the ChvE protein is also essential, suggesting that maximum *vir* gene induction is important (Heath et al., 1997).

The final chapter in the *Agrobacterium* transformation and regeneration of a whole host of cereals came from a series of papers from Japan Tobacco Inc. These highly significant papers make it abundantly clear that successful transformation and subsequent regeneration requires the collaboration of numerous investigators skilled in plant biology, tissue culture, and agrobiology (Hiei et al., 1994; Ishida et al., 1996; Gelvin, 2008).

Remarkably, the host range of *Agrobacterium* extends well beyond plants. In the laboratory, representative algae (Kumar et al., 2004), fungi (Bundock et al., 1995), and even human cells (Kunik et al., 2001) have all been infected. Additional non-plant organisms transformed by *Agrobacterium* are reviewed in Soltani et al. (2008). These expanded abilities now allow the revolution in plant molecular biology and genetics that *Agrobacterium* made possible in plants to be extended to a whole host of other eukaryotic systems.

## RELEVANT TOPICS NOT COVERED

Restrictions on the length of this review preclude discussions of many aspects of crown gall tumorigenesis which deserve attention. One such area is the story of the journey of the T-strand, once inside the host cell, to its site of integration and the plant factors involved. Fortunately, several recent reviews cover this aspect (Gelvin, 2010a,b, 2012; Lacroix and Citovsky, 2013).

Another area of great interest and importance in a discussion of plant tumorigenesis is that of plant defense against *Agrobacterium*. This subject has also been covered recently (Pitzschke, 2013).

A third aspect of great importance in the interaction of *Agrobacterium* with its hosts relates to the two-way signal exchange between *Agrobacterium* and its host plants. A recently published review covers many aspects of this subject (Subramoni et al., 2014).

Another inadequately covered area in this review is that of *Agrobacterium* and its role in plant biotechnology. This subject was covered in an excellent review (Banta and Montenegro, 2008).

The subject of horizontal gene transfer from *Agrobacterium* to plants in nature is an intriguing subject. This subject was recently reviewed (Matveeva and Lutova, 2014).

My apologies to the many crown gall investigators whose contributions I have not been able to cover or their studies cited because of space limitations.

## SUMMARY

It is unusual that the study of a single organism can reveal a unique biological system, contribute to an understanding of fundamental biological principles and lead to the development of an entirely new industry. In addition, *Agrobacterium* has revolutionized plant molecular genetics and has the capabilities to do the same for many other eukaryotic organisms. Certainly, Smith and Townsend (1907) studying the cause of a destructive plant disease and perhaps even Armin Braun who recognized the unique character of this disease could not have imagined how exciting and important the study of *Agrobacterium* and crown gall would turn out to be.

However, as much as we have learned about the mechanism by which *Agrobacterium* transforms plant cells, many features of this bacterium-host relationship deserve much more study. Clearly, we need to learn more about the world of *Agrobacterium* in its natural environment. What is the microbiome inside and outside the rhizosphere of a susceptible plant? What mechanisms does *Agrobacterium* use to successfully maintain its niche in the rhizosphere and what environmental cues and plant signals does it co-opt to promote its success? How does it recognize these signals? Does *Agrobacterium* send signals to the plant which promote the secretion of signals beneficial to *Agrobacterium*? What plant genes are affected by molecules secreted by the bacterium and do some contribute to host defense? What is the mechanism and consequences of attachment of *Agrobacterium* to plant cells? Does this affect the physiology of the bacterium? Answers to some of these questions may reveal the functions of some of the *chv* mutations which lead to attenuated virulence.

The natural environment of a tumor should also be studied with time-course studies of tumor development. Do the products of transformation, phytohormones and opines, modify the physiology of *Agrobacterium*? Is the *vir* regulon turned off in bacteria within a tumor? If so, what turns it off?

Another exciting area that should receive increasing attention is the detailed analysis of the travels of the T-DNA from the time *Agrobacterium* attaches to the host cell until the DNA becomes integrated into the host chromosome. Conflicting reports in some important aspects of this journey need to be resolved. The identification and availability of plant mutants will make such studies increasingly amenable to detailed analysis and understanding.

The study of *Agrobacterium*–plant interactions continues to point out just how clever this pathogen is. Future studies will no doubt reveal additional examples of how *Agrobacterium* takes advantage of its natural environment to further its own goals.

### Conflict of Interest Statement

The author declares that the research was conducted in the absence of any commercial or financial relationships that could be construed as a potential conflict of interest.

## References

[B1] Akiyoshi D. E. Klee H. Amasino R. M. Nester E. W. Gordon M. P. (1984). T-DNA of *Agrobacterium* encodes an enzyme of cytokinin biosynthesis. Proc. Natl. Acad. Sci. U.S.A. 81, 5994–5998. 10.1073/pnas.81.19.5994 6091129 PMC391845

[B2] Akiyoshi D. E. Morris R. O. Hinz R. Mische B. S. Kosuge T. Garfinkel D. J. (1983). Cytokinin/auxin balance in crown gall tumors is regulated by specific loci in the T-DNA. Proc. Natl. Acad. Sci. U.S.A. 80, 407–411. 10.1073/pnas.80.2.407 16593270 PMC393386

[B3] Albright L. Yanofsky M. F. Leroux B. Ma D. Nester E. W. (1987). Processing of the T-DNA of *Agrobacterium tumefaciens* generates border knicks and linear, single stranded T-DNA. J. Bacteriol. 169, 1046–1055. 3029014 10.1128/jb.169.3.1046-1055.1987 PMC211899

[B4] Allardet-Servent A. Michaux-Charachon S. Jumas-Bilak M. Karayan L. Ramuz M. (1993). Presence of one linear and one circular chromosome in the *Agrobacterium tumefaciens* C58 genome. J. Bacteriol. 175, 7869–7874. 8253676 10.1128/jb.175.24.7869-7874.1993 PMC206964

[B5] Alvarez-Martinez C. E. Christie P. J. (2009). Biological diversity of prokaryotic type IV secretion systems. Microbiol. Mol. Biol. Rev. 73, 775–808. 10.1128/MMBR.00023-09 19946141 PMC2786583

[B6] Ankenbauer R. G. Nester E. W. (1990). Sugar mediated induction of *Agrobacterium* virulence genes: structural specificity and activities of monosaccharides. J. Bacteriol. 172, 6442–6446. 2121715 10.1128/jb.172.11.6442-6446.1990 PMC526831

[B7] Atmakuri K. Cascales E. Burton O. T. Banta L. M. Christie P. J. (2007). *Agrobacterium* ParA/MinD-like VirC1 spatially coordinate early conjugative DNA transfer reactions. EMBO J. 26, 2540–2551. 10.1038/sj.emboj.7601696 17505518 PMC1868908

[B8] Ballas N. Citovsky V. (1997). Nuclear localization signal binding protein from *Arabidopsis* mediates nuclear import of *Agrobacterium* VirD2 protein. Proc. Natl. Acad. Sci. U.S.A. 94, 10723–10728. 10.1073/pnas.94.20.10723 9380702 PMC23464

[B9] Banta L. M. Joeger R. D. Horvitz V. R. Campbell A. M. Binns A. N. (1994). Glu-255 outside the predicted ChvE binding site in VirA is crucial for sugar enhancement of acetosyringone perception by *Agrobacterium*. J. Bacteriol. 176, 3242–3249. 8195079 10.1128/jb.176.11.3242-3249.1994 PMC205494

[B10] Banta L. M. Montenegro M. (2008). “*Agrobacterium* and plant biotechnology,” in Agrobacterium: From Biology to Biotechnology, eds Tzfira T. Citovsky V. (New York, NY: Springer Science+Business Media), 73–147.

[B11] Bhattacharjee S. Lee L. Oltmanns H. Cao H. Veena J. C. Gelvin S. B. (2008). IMPa-4, an *Arabidopsis* importin *α* isoform, is preferentially involved in *Agrobacterium*-mediated plant transformation. Plant Cell 20, 2661–2680. 10.1105/tpc.108.060467 18836040 PMC2590722

[B12] Binns A. N. (2005). “Armin C. Braun and the discovery of *Agrobacterium*-mediated transformation of plant cells,” in Agrobacterium tumefaciens: From Plant Pathology To Biotechnology, eds Nester E. Gordon M. P. Kerr A. (St. Paul, MN: APS Press), 7–10.

[B13] Blaha G. Stanley R. E. Steitz T. A. (2009). Formation of the first peptide bond: the structure of EF-P bound to the 70S ribosome. Science 325, 966–970. 10.1126/science.1175800 19696344 PMC3296453

[B14] Boulton M. I. Buchholz W. G. Marks M. S. Markam P. G. Davies J. (1989). Specificity of *Agrobacterium*-mediated delivery of maize streak virus DNA to members of the Gramineae. Plant Mol. Biol. 12, 31–40. 10.1007/BF00017445 24272715

[B15] Braun A. C. (1947). Thermal studies on tumor inception in the crown gall disease. Am. J. Bot. 30, 674–677.

[B16] Braun A. C. (1958). A physiological basis for autonomous growth of the crown-gall tumor cell. Proc. Natl. Acad. Sci. U.S.A. 44, 344–349. 10.1073/pnas.44.4.344 16590203 PMC335424

[B17] Braun A. C. Mandel R. J. (1948). Studies on the inactivation of the tumor inducing principle in crown gall. Growth 12, 14–28. 18107376

[B18] Brencic A. Eberhard A. Winans S. C. (2004). Signal quenching, detoxification and mineralization of vir gene-inducing phenolics by the VirH2 protein of *Agrobacterium tumefaciens*. Mol. Microbiol. 51, 1103–1115. 10.1046/j.1365-2958.2003.03887.x 14763983

[B19] Bundock P. denDulk-Ras A. Beijersbergen A. Hooykaas P. J. J. (1995). Trans-kingdom T-DNA transfer from *Agrobacterium tumefaciens* to *Saccharomyces cerevisiae*. EMBO J. 14, 3206–3214. 7621833 10.1002/j.1460-2075.1995.tb07323.x PMC394382

[B20] Bytebier B. Deboeck F. DeGreve H. Van Montagu M. Hernalsteens J. P. (1987). T-DNA organization in tissue culture and transgenic plants of the monocotyledon *Asparagus officinalis*. Proc. Natl. Acad. Sci. U.S.A. 83, 3282–3286. 10.1073/pnas.84.15.5345 PMC298852 16593862

[B21] Cangelosi G. A. Ankenbauer R. G. Nester E. W. (1990a). Sugars induce the *Agrobacterium* virulence genes through a periplasmic binding protein and a transmembrane signal protein. Proc. Natl. Acad. Sci. U.S.A. 87, 6708–6712. 10.1073/pnas.87.17.6708 2118656 PMC54606

[B22] Cangelosi, G, A., Martinetti, G., Nester E. W. (1990b). Osmosensitivity phenotypes of mutants of *Agrobacterium tumefaciens* that lack periplasmic beta-1,2 glucan. J. Bacteriol. 172, 2172–2174. 2318812 10.1128/jb.172.4.2172-2174.1990 PMC208718

[B23] Cascales E. Atmakuri K. Sakar M. K. Christie P. J. (2013). DNA substrate – induced activation of the *Agrobacterium* VirB/VirD4 type IV secretion system. J. Bacteriol. 195, 2691–2704. 10.1128/JB.00114-13 23564169 PMC3676061

[B24] Cascales E. Christie P. J. (2004). Definition of a bacterial type IV secretion pathway for a DNA substrate. Science 304, 1170–1173. 10.1126/science.1095211 15155952 PMC3882297

[B25] Chang C.-H. Winans S. C. (1992). Functional roles assigned to the periplasmic, linker and receiver domains of the *Agrobacterium tumefaciens* VirA protein. J. Bacteriol. 174, 7033–7039. 1400253 10.1128/jb.174.21.7033-7039.1992 PMC207384

[B26] Charles T. Jin S. Nester E. (1992). Two-component transduction systems in phytobacteria. Ann. Rev. Phytopathol. 30, 463–484 10.1146/annurev.py.30.090192.002335 18647108

[B27] Charles T. C. Nester E. W. (1993). A chromosomally-encoded two-component sensory transduction system is required for virulence of *Agrobacterium tumefaciens*. J. Bacteriol. 175, 6614–6625. 8407839 10.1128/jb.175.20.6614-6625.1993 PMC206773

[B28] Chateau S. Sangwan R. S. Sangwan-Norreel B. S. (2000). Competence of *Arabidopsis thaliana* genotypes and mutants for *Agrobacterium tumefaciens*-mediated gene transfer: role of phytohormones. J. Exp. Bot. 51, 1961–1968. 10.1093/jexbot/51.353.1961 11141170

[B29] Chen E. J. Fisher R. F. Perovich V. M. Sabio E. A. Long S. R. (2009). Identification of direct transcriptional target genes of ExoS/ChvI two-component signaling in *Sinorhizobium meliloti*. J. Bacteriol. 191, 6833–6842. 10.1128/JB.00734-09 19749054 PMC2772461

[B30] Chen E. J. Sabio E. A. Long S. R. (2008). The periplasmic regulator ExoR inhibits ExoS/ChvI two-component signaling in *Sinorhizobium meliloti*. Mol. Microbiol. 69, 1290–1303. 10.1111/j.1365-2958.2008.06362.x 18631237 PMC2652646

[B31] Chilton M.-D. Drummond M. H. Merlo D. J. Sciaky D. Montoya A. L. Gordon M. P. (1977). Stable incorporation of plasmid DNA into higher plant cells: the molecular basis of crown gall tumorigenesis. Cell 11, 263–271 10.1016/0092-8674(77)90043-5 890735

[B32] Chilton M.-D. Montoya A. L. Merlo D. J. Drummond M. H. Nutter R. Gordon M. P. (1978). Restriction endonuclease mapping of a plasmid that confers oncogenicity upon *Agrobacterium tumefaciens* strain B6-806. Plasmid 1, 254–269 10.1016/0147-619X(78)90043-4 748950

[B33] Christie P. J. Ward J. E. Winans S. C. Nester E. W. (1988). The *Agrobacterium tumefaciens* gene product VirE2 is a single-stranded DNA-binding protein that associates with T-DNA. J. Bacteriol. 170, 2659–2667. 2836366 10.1128/jb.170.6.2659-2667.1988 PMC211185

[B34] Citovsky V. DeVos G. Zambryski P. (1988). Single-stranded DNA binding protein encoded by the virE2 locus of *Agrobacterium*. Science 240, 501–504. 10.1126/science.240.4851.501 17784072

[B35] Das A. (1988). *Agrobacterium tumefaciens* virE operon encodes a single-stranded DNA binding protein. Proc. Natl. Acad. Sci. U.S.A. 85, 2909–2913. 10.1073/pnas.85.9.2909 2452439 PMC280112

[B36] Das A. Stachel S. Ebert P. Allenza P. Montoya A. Nester E. (1986). Promoters of *Agrobacterium tumefaciens* Ti-plasmid virulence genes. Nucleic Acids Res. 14, 1355–1364. 10.1093/nar/14.3.1355 3513123 PMC339509

[B37] DeCleene M. DeLey J. (1976). The host range of crown gall. Bot. Rev. 42, 389–466.

[B38] Deng W. Chen L. Peng W.-T. Metcalf T. T. Liang X. Gordon M. P. (1999). Vir E1 is a specific molecular chaperone for the exported single-stranded-DNA-binding protein VirE2 in *Agrobacterium*. Mol. Microbiol. 31, 1795–1807. 10.1046/j.1365-2958.1999.01316.x 10209751

[B39] Doty S. L. Yu M. C. Lundin J. I. Heath J. D. Nester E. W. (1996). Mutational analysis of the input domain of the VirA protein of *Agrobacterium tumefaciens*. J. Bacteriol. 178, 961–970. 8576069 10.1128/jb.178.4.961-970.1996 PMC177754

[B40] Douglas C. J. Halperin W. Nester E. W. (1982). *Agrobacterium tumefaciens* mutants affected in attachment to plant cells. J. Bacteriol. 152, 1265–1275. 6292165 10.1128/jb.152.3.1265-1275.1982 PMC221638

[B41] Douglas C. J. Staneloni R. J. Rubin R. A. Nester E. W. (1985). Identification and genetic analysis of an *Agrobacterium tumefaciens* chromosomal virulence region. J. Bacteriol. 161, 850–860. 2982791 10.1128/jb.161.3.850-860.1985 PMC214975

[B42] Drummond M. H. Gordon M. P. Nester E. W. Chilton M.-D. (1977). Foreign DNA of bacterial origin is transcribed in crown gall tumors. Nature 269, 535–536 10.1038/269535a0

[B43] Durrenberger F. Crameri A. Hohn B. Koukolikova-Nicola Z. (1989). Covalently bound VirD2 protein of *Agrobacterium tumefaciens* protects the T-DNA from exonucleolytic degradation. Proc. Natl. Acad. Sci. U.S.A. 86, 9154–9158. 10.1073/pnas.86.23.9154 2556703 PMC298452

[B44] Dym O. Albeck S. Unger T. Jacobovitch J. Branz-burg A. Michael Y. (2008). Crystal structure of the *Agrobacterium* virulence complex VirE1-VirE2 reveals a flexible protein that can accomodate different partners. Proc. Natl. Acad. Sci. U.S.A. 105, 11170–11175. 10.1073/pnas.0801525105 18678909 PMC2516231

[B45] Fullner K. J. Lara J. C. Nester E. W. (1996). Pilus assembly by *Agrobacterium* T-DNA transfer genes. Science 273, 1107–1109. 10.1126/science.273.5278.1107 8688097

[B46] Garfinkel D. Nester E. W. (1980). *Agrobacterium tumefaciens* mutants affected in crown gall tumorigenesis and octopine catabolism. J. Bacteriol. 144, 732–743. 6253441 10.1128/jb.144.2.732-743.1980 PMC294723

[B47] Garfinkel D. J. Simpson R. P. Ream L. W. White F. F. Gordon M. P. Nester E. W. (1981). Genetic analysis of crown gall: fine structure map of the T-DNA by site-directed mutagenesis. Cell 27, 143–15323 10.1016/0092-8674(81)90368-8 6276020

[B48] Gelvin S. B. (1998). *Agrobacterium* VirE2 protein can form a complex with T-strands in the plant cytoplasm. J. Bacteriol. 180, 4300–4302. 9696783 10.1128/jb.180.16.4300-4302.1998 PMC107431

[B49] Gelvin S. B. (2003). *Agrobacterium*-mediated plant transformation: the biology behind the “gene-jockeying tool.” Microbiol. Mol. Bio. Rev. 67, 16–37 10.1128/MMBR.67.1.16-37.2003 12626681 PMC150518

[B50] Gelvin S. B. (2008). “Function of host proteins in the *Agrobacterium*-mediated plant transformation process,” in Agrobacterium: From Biology to Biotechnology, eds Tzfira T. Citovsky V. (New York, NY: Springer Science+Business Media), 489–522.

[B51] Gelvin S. B. (2010a). Finding a way to the nucleus. Curr. Opin. Microbiol. 13, 53–58. 10.1016/j.mib.2009.11.003 20022799

[B52] Gelvin S. B. (2010b). Plant proteins involved in *Agrobacterium*-mediated genetic transformation. Annu. Rev. Phytopathol. 48, 45–68. 10.1146/annurev-phyto-080508-081852 20337518

[B53] Gelvin S. B. (2012). Traversing the cell: *Agrobacterium* T-DNA’s journey to the host genome. Front. Plant Sci. 3:52. 10.3389/fpls.2012.00052 22645590 PMC3355731

[B54] Gelvin S. B. Gordon M. P. Nester E. W. Aronson A. I. (1981). Transcription of *Agrobacterium* Ti-plasmid in the bacterium and crown gall tumors. Plasmid 6, 17–29 10.1016/0147-619X(81)90051-2 7280084

[B55] Gelvin S. B. Thomashow M. F. McPherson J. C. Gordon M. P. Nester E. W. (1982). Sizes and map positions of T-DNA encoded transcripts in octopine-type crown gall tumors. Proc. Natl. Acad. Sci. U.S.A. 79, 76–80. 10.1073/pnas.79.1.76 6275392 PMC345664

[B56] Gietl C. Koukolikova-Nicola Z. Hohn B. (1987). Mobilization of T-DNA from *Agrobacterium* to plant cells involves a protein that binds single-stranded DNA. Proc. Natl. Acad. Sci. U.S.A. 84, 9006–9010. 10.1073/pnas.84.24.9006 3480525 PMC299680

[B57] Goodner B. Hinkle G. Gattung S. Miller N. Blanchard M. Qurollo B. (2001). Genome sequence of the plant pathogen and biotechnology agent *Agrobacterium tumefaciens* C58. Science 294, 2323–2328. 10.1126/science.1066803 11743194

[B58] Gray J. Gelvin S. B. Meilan R. Morris R. O. (1996). Transfer RNA is the source of extracellular isopentenyladenine in a Ti-plasmidless strain of *Agrobacterium tumefaciens*. Plant Physiol. 110, 431–438. 12226194 10.1104/pp.110.2.431 PMC157737

[B59] Gray J. Wang J. Gelvin S. B. (1992). Mutation of the *miaA* gene of *Agrobacterium tumefaciens* results in reduced vir gene expression. J. Bacteriol. 174, 1086–1098. 1735704 10.1128/jb.174.4.1086-1098.1992 PMC206401

[B60] Grimsley N. Hohn B. Hohn T. Walden R. (1986). Agroinfection, a novel route for plant viral infection using Ti plasmid. Proc. Natl. Acad. Sci. U.S.A. 83, 3282–3286. 10.1073/pnas.83.10.3282 16593697 PMC323497

[B61] Grimsley N. Hohn T. Davies J. W. Hohn B. (1987). *Agrobacterium*-mediated delivery of infectious maize streak virus into maize plants. Nature 325, 177–179 10.1038/325177a0

[B62] Hamilton R. H. (2005). “Loss of tumor-inducing ability,” in Agrobacterium tumefaciens: From Plant Pathology to Biotechnology, eds Nester E. W. Gordon M. P. Kerr A. (St. Paul, MN: APS Press), 45–46.

[B63] Hamilton R. H. Fall M. Z. (1971). The loss of tumor-initiating ability in *Agrobacterium tumefaciens* by incubation at high temperature. Experentia 27, 229–230 10.1007/BF02145913 5544763

[B64] He F. Nair G. R. Soto C. S. Chang Y. Hsu L. Ronzone E. (2009). Molecular basis of ChvE function in sugar binding, sugar utilization and virulence in *Agrobacterium tumefaciens*. J. Bacteriol. 191, 5802–5813. 10.1128/JB.00451-09 19633083 PMC2737963

[B65] Heath J. D. Boulton M. I. Raineri D. M. Doty S. L. Mushegian A. R. Charles T. C. (1997). Discrete regions of the sensor protein VirA determine the strain specific ability of *Agrobacterium* to infect maize. Mol. Plant Microbe Interact. 10, 221–227. 10.1094/MPMI.1997.10.2.221 9057328

[B66] Heckel B. C. Tomlinson A. D. Morton E. R. Choi J.-H. Fuqua C. (2014). *Agrobacterium tumefaciens* ExoR controls acid response genes and impacts exopolysaccharide synthesis, horizontal gene transfer and virulence gene expression. J. Bacteriol. 196, 3221–3233. 10.1128/JB.01751-14 24982308 PMC4135700

[B67] Herrera-Estrella A. Van Montagu M. Wang K. (1990). A bacterial peptide acting as a nuclear targeting signal: the amino terminal portion of *Agrobacterium* VirD2 protein directs a *β*-galactosidase fusion protein into tobacco nuclei. Proc. Natl. Acad. Sci. U.S.A. 87, 9534–9537. 10.1073/pnas.87.24.9534 2124696 PMC55206

[B68] Hersch S. J. Wang M. Zou S. B. Moon K.-M. Foster L. J. Ibba M. (2013). Divergent protein motifs direct elongation factor P-mediated translational regulation in *Salmonella enterica* and *Escherichia coli*. MBio 4, 1–10. 10.1128/mBio.00180-13 23611909 PMC3638311

[B69] Hiei Y. Ohta S. Komari T. Kumashiro T. (1994). Efficient transformation of rice (*Oryza sativa* L.) mediated by *Agrobacterium* and sequence analysis of the boundaries of the T-DNA. Plant J. 6, 271–282. 10.1046/j.1365-313X.1994.6020271.x 7920717

[B70] Holsters M. Silva B. Van Vleit F. Genetello C. De Block M. Dhaese P. (1980). The functional organization of the nopaline *A. tumefaciens* plasmid pTiC58. Plasmid 3, 212–230 10.1016/0147-619X(80)90110-9 6100894

[B71] Hood E. E. Helmer G. L. Fraley R. T. Chilton M.-D. (1986). The hypervirulence of *Agrobacterium tumefaciens* A281 is encoded in a region of pTiBo542 outside of T-DNA. J. Bacteriol. 168, 1291–1301. 3782037 10.1128/jb.168.3.1291-1301.1986 PMC213636

[B72] Howard E. A. Zupan J. R. Citovsky V. Zambryski P. C. (1992). The VirD2 protein of *A. tumefaciens* contains a C-terminal bipartite nuclear localization signal: implications for nuclear uptake of DNA in plant cells. Cell 68, 109–118 10.1016/0092-8674(92)90210-4 1732061

[B73] Hu X. Zhao J. DeGrado W. F. Binns A. N. (2013). *Agrobacterium tumefaciens* recognizes its host plant environment using ChvE to bind diverse plant sugars as virulence signals. Proc. Natl. Acad. Sci. U.S.A. 110, 678–683. 10.1073/pnas.1215033110 23267119 PMC3545744

[B74] Hwang H.-H. Wang M.-H. Lee Y.-L. Tsai Y. L. Li Y.-H. Yang F. H. (2010). *Agrobacterium*-produced and exogenous cytokinin-modulated *Agrobacterium*-mediated plant transformation. Mol. Plant Pathol. 11, 677–690. 10.1111/j.1364-3703.2010.00637.x 20696005 PMC6640272

[B75] Ishida Y. Saito H. Ohta S. Hiei Y. Komari T. Kumashiro T. (1996). High efficiency transformation of maize (*Zea mays* L.) mediated by *Agrobacterium tumefaciens*. Nat. Biotechnol. 14, 745–750. 10.1038/nbt0696-745 9630983

[B76] Jia Y. H. Li L. P. Hou Q. M. Pan S. Q. (2002). An *Agrobacterium* gene involved in tumorigenesis encodes an outer membrane protein exposed on the bacterial cell surface. Gene 284, 113–124 10.1016/S0378-1119(02)00385-2 11891052

[B77] Jin S. Komari T. Gordon M. P. Nester E. W. (1987). Genes responsible for the supervirulent phenotype of *Agrobacterium tumefaciens* strain A281. J. Bacteriol. 169, 4417–4425. 2443480 10.1128/jb.169.10.4417-4425.1987 PMC213802

[B78] Jin S. Roitsch T. Ankenbauer R. G. Gordon M. P. Nester E. W. (1990a). The VirA protein of *Agrobacterium tumefaciens* is autophosphorylated and is essential for vir gene regulation. J. Bacteriol. 172, 525–530. 2404940 10.1128/jb.172.2.525-530.1990 PMC208473

[B79] Jin S. Prusti R. Roitsch T. Ankenbauer R. G. Nester E. W. (1990b). Phosphorylation of the VirG protein of *Agrobacterium tumefaciens* by the autophosphorylated VirA protein: essential role in biological activity of VirG. J. Bacteriol. 172, 4945–495042. 2394678 10.1128/jb.172.9.4945-4950.1990 PMC213149

[B80] Jin S. Roitsch T. Christie P. J. Nester E. W. (1990c). The regulatory VirG protein specifically binds to *cis*-acting regulatory sequence involved in transcriptional activation of *Agrobacterium tumefaciens* virulence genes. J. Bacteriol. 172, 531–537. 2404941 10.1128/jb.172.2.531-537.1990 PMC208474

[B81] Johnson R. Guiderian R. H. Eden F. Chilton M.-D. Gordon M. P. Nester E. W. (1974). Detection and quantification of octopine in normal plant tissue and crown gall tumors. Proc. Natl. Acad. Sci. U.S.A. 71, 536–539. 16592142 10.1073/pnas.71.2.536 PMC388042

[B82] Kado C. (2014). Historical account on gaining insights on the mechanism of crown gall tumorigenesis induced by *Agrobacterium tumefaciens*. Front. Microbiol. 5:340. 10.3389/fmicb.2014.00340 25147542 PMC4124706

[B83] Kalogeraki V. S. Winans S. C. (1998). Wound released chemical signals may elicit multiple responses from an *Agrobacterium tumefaciens* strain containing an octopine-type Ti plasmid. J. Bacteriol. 180, 5660–5667. 9791116 10.1128/jb.180.21.5660-5667.1998 PMC107625

[B84] Kalogeraki V. S. Zhu J. Stryker J. L. Winans S. C. (2000). The right end of the vir region of an octopine-type Ti plasmid contains four new members of the vir regulon that are not required for pathogenesis. J. Bacteriol. 182, 1774–1778. 10.1128/JB.182.6.1774-1778.2000 10692388 PMC94480

[B85] Kanemoto R. H. Powell A. T. Akiyoshi D. E. Regier D. A. Kerstetter R. A. Nester E. W. (1989). Nucleotide sequence and analysis of the plant-inducible locus, pinF, from *Agrobacterium tumefaciens*. J. Bacteriol. 171, 2506–2512. 2708311 10.1128/jb.171.5.2506-2512.1989 PMC209927

[B86] Kemner J. M. Liang X. Nester E. W. (1997). The *Agrobacterium tumefaciens* virulence gene chvE is part of a putative ABC-type sugar transport operon. J. Bacteriol. 179, 2452–2458. 9079938 10.1128/jb.179.7.2452-2458.1997 PMC178989

[B87] Kerr A. (1969). Transfer of virulence between isolates of *Agrobacterium*. Nature 223, 1175–1176 10.1038/2231175a0

[B88] Kerr A. (2005). “Treasure the unexpected,” in Agrobacterium tumefaciens: From Plant Pathology to Biotechnology, eds Nester E. W. Gordon M. P. Kerr A. (St. Paul, MN: APS Press), 29–30.

[B89] Kerr J. E. Christie P. J. (2010). Evidence for VirB4-mediated dislocation of membrane-integrated VirB2 pilin during biogenesis of the *Agrobacterium* VirB/VirD4 type IV secretion system. J. Bacteriol. 192, 4923–4934. 10.1128/JB.00557-10 20656905 PMC2944537

[B90] Klee H. Montoya A. Horodyski F. Lichtenstein C. Garfinkel D. Fuller S. (1984). Nucleotide sequences of the *tms* genes of the pTiA6NC octopine Ti-plasmid: two gene products involved in plant tumorigenesis. Proc. Natl. Acad. Sci. U.S.A. 81, 1728–1732. 10.1073/pnas.81.6.1728 6584906 PMC344992

[B91] Kumar S. V. Misquitta R. W. Reddy V. S. Rao B. J. Rajam M. V. (2004). Genetic transformation of the green alga *Chlamydomonas reinhardtii* by *Agrobacterium tumefaciens*. Plant Sci. 166, 731–738 10.1016/j.plantsci.2003.11.012

[B92] Kunik T. Tzfira T. Kapulnik Y. Gafni Y. Dingwall C. Citovsky V. (2001). Genetic transformation of HeLa cells by *Agrobacterium*. Proc. Natl. Acad. Sci, U.S.A. 98, 1871–1876. 10.1073/pnas.98.4.1871 11172043 PMC29349

[B93] Lacroix B. Citovsky V. (2013). The roles of bacterial and host plant factors in *Agrobacterium*-mediated genetic transformation. Int. J. Dev. Biol. 57, 467–481. 10.1387/ijdb.130199bl 24166430 PMC9478875

[B94] Lacroix B. Vaidya M. Tzfira T. Citovsky V. (2005). The VirE3 protein of *Agrobacterium* mimics a host cell function required for plant genetic transformation. EMBO J. 24, 428–437. 10.1038/sj.emboj.7600524 15616576 PMC545813

[B95] Lai E.-M. Kado C. I. (1998). Processed VirB2 is the major subunit of the promiscuous pilus of *Agrobacterium tumefaciens*. J. Bacteriol. 180, 2711–2717. 9573157 10.1128/jb.180.10.2711-2717.1998 PMC107224

[B96] Lai E.-M. Shih H.-W. Wen S.-W. Cheng M.-W. Hwang H.-H. Chiu S.-H. (2006). Proteomic analysis of *Agrobacterium tumefaciens* response to the vir gene inducer acetosyringone. Proteomics 6, 4130–4136 10.1002/pmic.200600254 16791832

[B97] Lee Y.-W. Jin S. Sim W.-S. Nester E. W. (1995). Genetic evidence for direct sensing of phenolic compounds by the VirA protein of *Agrobacterium tumefaciens*. Proc. Natl. Acad. Sci., U.S.A. 92, 12245–12249. 8618878 10.1073/pnas.92.26.12245 PMC40333

[B98] Lemmers M. Holsters M. Zambryski P. DePicker A. Hernalsteens J. P. Van Montagu M. (1980). Internal organization, boundaries and integration of Ti-plasmid DNA in nopaline crown gall tumors. J. Mol. Biol. 144, 353–376 10.1016/0022-2836(80)90095-9 7253020

[B99] Leroux B. Yanofsky M. F. Winans S. C. Ward J. E. Ziegler S. F. Nester E. W. (1987). Characterization of the virA locus of *Agrobacterium tumefaciens*: a transcriptional regulator and host range determinant. EMBO J. 6, 849–856. 3595559 10.1002/j.1460-2075.1987.tb04830.x PMC553474

[B100] Li L. Jia Y. Hori Q. Charles T. C. Nester E. W. Pan S. (2002). A global pH sensor: *Agrobacterium* sensor protein ChvG regulates acid-inducible genes on its two chromosomes and Ti plasmid. Proc. Natl. Acad. Sci. U.S.A. 99, 12369–12374. 12218184 10.1073/pnas.192439499 PMC129451

[B101] Lioret C. (1957). Les acides amines’ libres de tissues de crown-gall. Mise en évidence d’un acide aminé particulier à ces tissues. C. R. Hebd. Seances Acad. Sci. 244, 2171–2174.

[B102] Liu P. Wood D. W. Nester E. W. (2005). Phosphoenolpyruvate carboxykinase is an acid induced, chromosomally encoded, virulence factor in *Agrobacterium tumefaciens*. J. Bacteriol. 187, 6039–6045. 10.1128/JB.187.17.6039-6045.2005 16109945 PMC1196135

[B103] Liu Z. Jacobs M. Schaff D. A. McCullen C. A. Binns A. N. (2001). ChvD, a chromosomally encoded ATP binding cassette transporter-homologous protein involved in regulation of virulence gene expression in *Agrobacterium tumefaciens*. J. Bacteriol. 183, 3310–3317. 10.1128/JB.183.11.3310-3317.2001 11344138 PMC99628

[B104] Low H. H. Gubellini F. Rivera-Calzada A. Braun N. Connery S. Dujeancourt A. (2014). Structure of a type IV secretion system. Nature 508, 550–553. 10.1038/nature13081 24670658 PMC3998870

[B105] Lu H.-X. Luo L. Yang M.-H. Cheng H.-P. (2012). *Sinorhizobium meliloti* ExoR is the target of periplasmic proteolysis. J. Bacteriol. 194, 4029–4040. 10.1128/JB.00313-12 22636773 PMC3416547

[B106] Ma L. S. Hachani A. Lin J. S. Filloux A. Lai E. M. (2014). *Agrobacterium tumefaciens* deploys a superfamily of type VI secretion DNase effectors as weapons for interbacterial competition in plants. Cell Host Microbe 16, 94–104. 10.1016/j.chom.2014.06.002 24981331 PMC4096383

[B107] Magori S. Citovsky V. (2011). *Agrobacterium* counteracts host-induced degradation of its effector F-box protein. Sci. Signal. 4, ra69. 10.1126/scisignal.2002124 22009152 PMC9472992

[B108] Mantis N. J. Winans S. C. (1992). The *Agrobacterium* vir gene transcriptional activator VirG is transcriptionally induced by acid pH and other stress stimuli. J. Bacteriol. 174, 1189–1196. 1735712 10.1128/jb.174.4.1189-1196.1992 PMC206411

[B109] Mantis N. J. Winans S. C. (1993). The chromosomal response regulatory gene chvI of *Agrobacterium tumefaciens* complements an *Escherichia coli* phoB mutation and is required for virulence. J. Bacteriol. 175, 6626–6636. 8407840 10.1128/jb.175.20.6626-6636.1993 PMC206774

[B110] Matveeva T. V. Lutova L. A. (2014). Horizontal gene transfer from *Agrobacterium* to plants. Front. Plant Sci. 5:326. 10.3389/fpls.2014.00326 25157257 PMC4127661

[B111] Melchers L. S. Maroney M. J. den Dulk-Ras A. Thompson D. V. van Vuuren H. A. J. Schilperoort R. A. (1990). Octopine and nopaline strains of *Agrobacterium tumefaciens* differ in virulence; molecular characterization of the virF locus. Plant Mol. Biol. 14, 249–259. 10.1007/BF00018565 2101693

[B112] Moore L. Warren G. Strobel G. (1979). Involvement of a plasmid in the hairy root disease of plants. Plasmid 2, 617–626 10.1016/0147-619X(79)90059-3 231271

[B113] Mushegian A. R. Fullner K. J. Koonin E. V. Nester E. W. (1996). A family of lysozyme-like virulence factors in bacterial pathogens of plants and animals. Proc. Natl. Acad. Sci. U.S.A. 93, 7321–7326. 10.1073/pnas.93.14.7321 8692991 PMC38982

[B114] Nair G. R. Lai X. Wise A. Wonjae B. W. Jacobs M. Binns A. N. (2011). The integrity of the periplasmic domain of the VirA sensor kinase is critical for optimal coordination of the virulence signal response in *Agrobacterium tumefaciens*. J. Bacteriol. 193, 1436–1448. 10.1128/JB.01227-10 21216996 PMC3067612

[B115] Nixon B. T. Ronson C. W. Ausubel F. M. (1986). Two-component regulatory systems responsive to environmental stimuli share strongly conserved domains with the nitrogen assimilation regulatory genes ntrB and ntrC. Proc. Natl. Acad. Sci. U.S.A. 83, 8278–8282 10.1073/pnas.83.20.7850 3020561 PMC386820

[B116] Ooms G. Hooykaas P. J. J. Noleman G. Schilperoort R. A. (1981). Crown gall tumors of abnormal morphology induced by *Agrobacterium tumefaciens* carrying mutated Ti plasmids: analysis of T-DNA functions. Gene 14, 33–50 10.1016/0378-1119(81)90146-3 6266929

[B117] Pan S. Q. Charles T. Jin S. Wu Z.-L. Nester E. W. (1993). Preformed dimeric state of the sensor protein VirA is involved in plant-*Agrobacterium* signal transduction. Proc. Natl. Acad. Sci. U.S.A. 90, 9939–9943. 10.1073/pnas.90.21.9939 8234338 PMC47688

[B118] Pan S. Q. Jin S. Boulton M. I. Hawes M. Gordon M. P. Nester E. W. (1995). An *Agrobacterium* virulence factor encoded by a Ti plasmid gene or a chromosomal gene is required for T-DNA transfer into plants. Mol. Microbiol. 17, 259–269. 10.1111/j.1365-2958.1995.mmi_17020259.x 7494475

[B119] Pansegrau W. Schoumacher F. Hohn B. Lanka E. (1993). Site-specific cleavage and joining of single-stranded DNA by ViirD2 protein of *Agrobacterium tumefaciens* Ti plasmids: analogy to bacterial conjugation. Proc. Natl. Acad. Sci. U.S.A. 90, 11538–11542. 10.1073/pnas.90.24.11538 8265585 PMC48019

[B120] Pantoja M. Chen L. Chen Y. Nester E. W. (2002). *Agrobacterium* type IV secretion is a two-step process in which export substrates associate with the virulence protein VirJ in the periplasm. Mol. Microbiol. 45, 1325–1335. 10.1046/j.1365-2958.2002.03098.x 12207700

[B121] Pelczar P. Kalck V. Gomez D. Hohn B. (2004). *Agrobacterium* proteins VirD2 and VirE2 mediate precise integration of synthetic T-DNA complexes in mammalian cells. EMBO Rep. 5, 632–637. 10.1038/sj.embor.7400165 15153934 PMC1299075

[B122] Peng W.-T. Banta L. M. Charles T. C. Nester E. W. (2001). The chvH locus of *Agrobacterium* encodes a homolog of an elongation factor involved in protein synthesis. J. Bacteriol. 183, 36–45. 10.1128/JB.183.1.36-45.2001 11114898 PMC94847

[B123] Peng W. T. Lee Y. W. Nester E. W. (1998). The phenolic recognition profiles of the *Agrobacterium tumefaciens* VirA protein are broadened by a high level of the sugar binding protein, ChvE. J. Bacteriol. 180, 5632–5638. 9791112 10.1128/jb.180.21.5632-5638.1998 PMC107621

[B124] Peralta E. G. Hellmis R. Ream W. (1986). Overdrive, a T-DNA transmission enhancer on the *A. tumefaciens* tumor-inducing plasmid. EMBO J. 5, 1137–1142. 15966101 10.1002/j.1460-2075.1986.tb04338.x PMC1166919

[B125] Petit A. Delhaye S. Tempé J. Morel G. (1970). Reserches sur les guanidines des tissues de crown gall. Mise en évidence d’une relation biochemique spécifique entre les souches d’*Agrobacterium tumefaciens* et les tumeurs qu’eles induisent. Physiol. Vég. 8, 205–2174.

[B126] Pitzschke A. A. (2013). *Agrobacterium* and plant defense-transformation success hangs by a thread. Front. Plant Sci. 4: 519 10.3389/fpls.2013.00519 24391655 PMC3866890

[B127] Raineri D. M. Boulton M. I. Davies J. W. Nester E. W. (1993). VirA, the plant signal receptor, is responsible for the strain-specific transfer of DNA to maize by *Agrobacterium*. Proc. Natl. Acad. Sci. U.S.A. 90, 3549–3553. 10.1073/pnas.90.8.3549 8475103 PMC46338

[B128] Regensburg-Tuink A. J. Hooykaas P. J. J. (1993). Transgenic *N. glauca* plants expressing bacterial virulence gene virF are converted into hosts for nopaline strains of *A. tumefaciens*. Nature 363, 69–71. 10.1038/363069a0 8479538

[B129] Regier D. A. Morris R. O. (1982). Secretion of *trans*-zeatin by *Agrobacterium tumefaciens*: a function determined by the nopaline Ti-plasmid. Biochem. Biophys. Res. Commun. 104, 1560–1566 10.1016/0006-291X(82)91429-2 7073755

[B130] Ronson, C, V., Nixon, B. T., Ausubel F. M. (1987). Conserved domains in bacterial regulatory proteins that respond to environmental stimuli. Cell 49, 579–581 10.1016/0092-8674(87)90530-7 3555840

[B131] Rossi L. Hohn B. Tinland B. (1996). Integration of complete transferred T-DNA units is dependent on the activity of virulence E2 proteins of *Agrobacterium tumefaciens*. Proc. Natl. Acad. Sci. U.S.A. 93, 126–130. 10.1073/pnas.93.1.126 8552588 PMC40191

[B132] Russell A. B. Peterson S. B. Mougous J. D. (2014). Type VI secretion system effectors: poisons with a purpose. Nat. Rev. Microbiol. 12, 137–148. 10.1038/nrmicro3185 24384601 PMC4256078

[B133] Sakalis P. A. van Heusden G. P. H. Hooykaas P. J. J. (2014). Visualization of VirE2 protein translocation by the *Agrobacterium* type IV secretion system into host cells. Microbiol. Open 3, 104–117. 10.1002/mbo3.152 24376037 PMC3937733

[B134] Sardesai N. Lee L.-Y. Chen H. Yi H. Olbricht R. Stinberg A. (2013). Cytokinins secreted by *Agrobacterium* promote transformation by repressing a plant Myb transcription factor. Sci. Signal. 6, 1–11. 10.1126/scisignal.2004518 24255177

[B135] Seitz E. W. Hochster R. M. (1964). Lysopine in normal and in crown-gall tumor tissue of tomato and tobacco. Can. J. Bot. 42, 999–1004 10.1139/b64-091

[B136] Shi Y. Lee L.-Y. Gelvin S. B. (2014). Is VIP1 important for *Agrobacterium*-mediated transformation? Plant J. 79, 848–860. 10.1111/tpj.12596 24953893

[B137] Shimoda N. Toyoda-Yamamoto A. Aoki S. Machida Y. (1993). Genetic evidence for an interaction between the VirA sensor protein and the ChvE sugar-binding protein of *Agrobacterium*. J. Biol. Chem. 268, 26552–26558. 8253785

[B138] Shimoda N. Toyoda-Yamamoto A. Nagamine J. Usami S. Katayama M. Sakagami Y. (1990). Control of expression of *Agrobacterium vir* genes by synergistic actions of phenolic signal molecules and monosaccharides. Proc. Natl. Acad. Sci. U.S.A. 87, 6684–6688. 10.1073/pnas.87.17.6684 11607097 PMC54601

[B139] Smith E. F. Townsend C. O. (1907). A plant tumor of bacterial origin. Science 25, 671–673. 10.1126/science.25.643.671 17746161

[B140] Soltani J. van Heusden G. P. H. Hooykaas P. J. J. (2008). “*Agrobacterium*-mediated transformation of non-plant organisms,” in Agrobacterium: From Biology to Biotechnology, eds Tzfira T. Citovsky V. (New York, NY: Springer Science+Business Media), 649–675.

[B141] Stachel S. E. An G. Flores C. Nester E. W. (1985a). A Tn3 lacZ transposon for the random generation of *β*-galactosidase gene fusions: application to the analysis of gene expression in *Agrobacterium*. EMBO J. 4, 277–284. 2990912 10.1002/j.1460-2075.1985.tb03715.x PMC554276

[B142] Stachel S. E. Messens E. Van Montagu M. Zambryski P. (1985b). Identification of the signal molecules produced by wounded plant cells that activate T-DNA transfer in *Agrobacterium tumefaciens*. Nature 318, 624–628 10.1038/318624a0

[B143] Stachel S. E. Nester E. W. (1986). The genetic and transcriptional organization of the vir region of the A6 Ti-plasmid of *Agrobacterium tumefaciens*. EMBO J. 5, 1445–1454. 3017694 10.1002/j.1460-2075.1986.tb04381.x PMC1166964

[B144] Stachel S. E. Nester E. W. Zambryski P. (1986a). A plant cell factor induces *Agrobacterium tumefaciens vir* gene expression. Proc. Natl. Acad. Sci. U.S.A. 83, 379–383. 10.1073/pnas.83.2.379 16593648 PMC322862

[B145] Stachel S. E. Timmerman B. Zambryski P. (1986b). Generation of single-stranded T-DNA molecules during the initial stages of T-DNA transfer from *Agrobacterium tumefaciens* to plant cells. Nature 322, 706–712 10.1038/322706a0

[B146] Stachel S. E. Zambryski P. C. (1986). VirA and VirG control the plant induced activation of the T-DNA transfer process of *A. tumefaciens*. Cell 46, 325–333 10.1016/0092-8674(86)90653-7 3731272

[B147] Subramoni S. Nathoo N. Klimov E. Yuan Z.-C. (2014). *Agrobacterium tumefaciens* responses to plant-derived signaling molecules. Front. Plant Sci. 5: 322. 10.3389/fpls.2014.00322 25071805 PMC4086400

[B148] Sundberg C. D. Meek L. Carroll K. Das A. Ream W. (1996). VirE1 protein mediates export of the single-stranded DNA binding protein VirE2 from *Agrobacterium tumefaciens* into plant cells. J. Bacteriol. 178, 1207–1212. 8576060 10.1128/jb.178.4.1207-1212.1996 PMC177787

[B149] Suksomtip M. Liu P. Wood D. Nester E. W. (2005). Citrate synthase mutants of *Agrobacterium* are attenuated in virulence and display reduced *vir* gene induction. J. Bacteriol. 187, 4844–4852. 15995199 10.1128/JB.187.14.4844-4852.2005 PMC1169492

[B150] Thomashow L. S. Reeves S. Thomashow M. F. (1984). Crown gall oncogenesis: evidence that a T-DNA gene from the *Agrobacterium* Ti plasmid pTiA6 encodes an enzyme that catalyzes synthesis of indole-3-acetic acid. Proc. Natl. Acad. Sci. U.S.A. 81, 5071–5075. 10.1073/pnas.81.16.5071 6089175 PMC391639

[B151] Thomashow M. F. Hughly S. Buchlolz W. G. Thomashow L. S. (1986). Molecular basis for the auxin-independent phenotype of crown gall tumor tissue. Science 231, 616–618. 10.1126/science.3511528 3511528

[B152] Thomashow M. F. Nutter R. Montoya A. L. Gordon M. P. Nester E. W. (1980). Integration and organization of Ti plasmid sequences in crown gall tumors. Cell 19, 729–739 10.1016/S0092-8674(80)80049-3 7363328

[B153] Tinland B. Schoumacher F. Gloeckler V. Bravo-Angel A. M. Hohn B. (1995). The *Agrobacterium tumefaciens* virulence D2 protein is responsible for precise integration of T-DNA into the plant genome. EMBO J. 14, 3585–3595. 7628458 10.1002/j.1460-2075.1995.tb07364.x PMC394426

[B154] Toro N. Datta A. Carmi O. A. Young C. Prusti R. K. Nester E. W. (1989). The *Agrobacterium tumefaciens* virC1 gene product binds to overdrive, a T-DNA transfer enhancer. J. Bacteriol. 171, 6845–6849. 2592351 10.1128/jb.171.12.6845-6849.1989 PMC210585

[B155] Toro N. Datta A. Yanofsky M. Nester E. (1988). Role of overdrive sequence in T-DNA border cleavage. Proc. Natl. Acad. Sci. U.S.A. 85, 8558–8562. 10.1073/pnas.85.22.8558 3186745 PMC282498

[B156] Tsai Y. L. Chaing Y. R. Wu C. F. Narberhaus F. Lai E. M. (2012). One out of four: HspL but no other heat shock protein of *Agrobacterium tumefaciens* acts as efficient virulence-promoting VirB8 chaperone. PLoS ONE 7:e49685 10.1371/journal.pone.0049685 23185409 PMC3504140

[B157] Tzfira T. Vaidya M. Citovsky V. (2001). VIP1, an *Arabidopsis* protein that interacts with *Agrobacterium* VirE2, is involved in VirE2 nuclear import and *Agrobacterium* infectivity. EMBO J. 20, 3596–3607. 10.1093/emboj/20.13.3596 11432846 PMC125502

[B158] Tzfira T. Vaidya M. Citovsky V. (2004). Involvement of targeted proteolysis in plant genetic transformation by *Agrobacterium*. Nature 431, 87–92. 15343337 10.1038/nature02857

[B159] Van Larabeke N. Schell J. Schilperoort R. A. Hermans A. K. Hernalsteens J.-P. Montagu M. (1975). Acquisition of tumor-inducing ability by non-oncogenic agrobacteria as a result of plasmid transfer. Nature 255, 742–743 10.1038/255742a0 1134573

[B160] Veluthambi K. Ream W. Gelvin S. B. (1988). Virulence genes, borders, and overdrive generate single-stranded T-DNA molecules from the A6 Ti plasmid of *Agrobacterium tumefaciens*. J. Bacteriol. 170, 1523–1532. 2832367 10.1128/jb.170.4.1523-1532.1988 PMC210997

[B161] Ward E. R. Barnes W. M. (1988). VirD2 protein of *Agrobacterium tumefaciens* very tightly linked to the 5^′^ end of T-strand DNA. Science 242, 927–930 10.1126/science.242.4880.927

[B162] Ward J. E. Akiyoshi D. E. Regier D. Datta A. Gordon M. P. Nester E. W. (1988). Characterization of the virB operon from an *Agrobacterium tumefaciens* Ti plasmid. J. Biol. Chem. 263, 5804–5814. 3281947

[B163] Watson B. Currier T. C. Gordon M. P. Chilton M.-D. Nester E. W. (1975). Plasmid required for virulence of *Agrobacterium tumefaciens*. J. Bacteriol. 123, 255–264. 1141196 10.1128/jb.123.1.255-264.1975 PMC235714

[B164] Watson B. Nester E. W. (2005). “A plasmid was there after all,” in Agrobacterium tumefaciens: From Plant Pathology to Biotechnology, eds Nester E. W. Gordon M. P. Kerr A. (St. Paul, MN: APS Press), 53–55.

[B165] White F. F. Nester E. W. (1980). Relationships of plasmids responsible for hairy root and crown gall tumorigenicity. J. Bacteriol. 144, 710–720. 7430069 10.1128/jb.144.2.710-720.1980 PMC294721

[B166] White P. R. Braun A. C. (1941). Crown gall production by bacteria-free tumor tissues. Science 94, 239–241. 10.1126/science.94.2436.239 17745128

[B167] Willmitzer L. Simons G. Schell J. (1982). The TL-DNA in octopine crown gall tumors codes for seven well-defined polyadenylated transcripts. EMBO J. 1, 139–146. 16453403 10.1002/j.1460-2075.1982.tb01137.x PMC553008

[B168] Winans S. C. Ebert P. R. Stachel S. E. Gordon M. P. Nester E. W. (1986). A gene essential for *Agrobacterium* virulence is homologous to a family of positive regulatory loci. Proc. Natl. Acad. Sci. U.S.A. 83, 8278–8282. 10.1073/pnas.83.21.8278 3022288 PMC386911

[B169] Winans S. C. Kerstetter R. A. Nester E. W. (1988). Transcriptional regulation of the *virA* and *virG* genes of *Agrobacterium tumefaciens*. J. Bacteriol. 170, 4047–4054. 2842300 10.1128/jb.170.9.4047-4054.1988 PMC211408

[B170] Wirawan I. G. P. Kang H. W. Kojima M. (1993). Isolation and characterization of a chromosomal virulence gene of *Agrobacterium tumefaciens*. J. Bacteriol. 175, 3208–3212. 8491736 10.1128/jb.175.10.3208-3212.1993 PMC204646

[B171] Wise A. A. Fang F. Lin Y.-H. He F. Lynn D. Binns A. N. (2010). The receiver domain of hybrid histidine kinase VirA: an enhancing for gene expression in *Agrobacterium tumefaciens*. J. Bacteriol. 192, 1534–1542. 10.1128/JB.01007-09 20081031 PMC2832513

[B172] Wood D. W. Setubal J. C. Kaul R. Monks D. E. Kitajima J. P. Okura V. K. (2001). The genome of the natural genetic engineer *Agrobacterium tumefaciens* C58. Science 294, 2317–2323. 10.1126/science.1066804 11743193

[B173] Wu C.-F. Lin J.-S. Shaw G.-C. Lai E.-M. (2012). Acid-induced type VI secretion system is regulated by ExoR-ChvG/ChvI signaling cascade in *Agrobacterium tumefaciens*. PLOS Pathog. 8:e1002938. 10.1371/journal.ppat.1002938 23028331 PMC3460628

[B174] Xu X. Q. Pan S. Q. (2000). An *Agrobacterium* catalase is a virulence factor involved in tumorigenesis. Mol. Microbiol. 35, 407–414. 10.1046/j.1365-2958.2000.01709.x 10652101

[B175] Yadav N. S. Vanderleyden J. Bennett D. R. Barnes W. M. Chilton M.-D. (1982). Short direct repeats flank the T-DNA on a nopaline Ti-plasmid. Proc. Natl. Acad. Sci. U.S.A. 79, 6322–6326. 10.1073/pnas.79.20.6322 16593241 PMC347113

[B176] Yanofsky M. Lowe B. Montoya A. Rubin R. Krul W. Gordon M. (1985). Molecular and genetic analysis of factors controlling host-range in *Agrobacterium tumefaciens*. Mol. Gen. Genet. 201, 237–246 10.1007/BF00425665

[B177] Yanofsky M. Porter S. C. Young C. Albright L. M. Gordon M. P. Nester E. W. (1986). The virD operon of *Agrobacterium tumefaciens* encodes a site-specific endonuclease. Cell 47, 471–477 10.1016/0092-8674(86)90604-5 3021341

[B178] Young C. Nester E. W. (1988). Association of the VirD2 protein with the 5^′^ end of T-strands of *Agrobacterium tumefaciens*. J. Bacteriol. 170, 3367–3374. 3403506 10.1128/jb.170.8.3367-3374.1988 PMC211303

[B179] Yuan Z. C. Liu P. Saenkham P. Kerr K. Nester E. W. (2008). Transcriptome profiling and functional analysis of *Agrobacterium tumefaciens* reveals a general conserved response to acidic conditions (pH 5.5) and a complex acid-mediated signaling involved in *Agrobacterium*–plant interactions. J. Bacteriol. 190, 494–507. 10.1128/JB.01387-07 17993523 PMC2223696

[B180] Yusibov V. M. Steck T. R. Gupta V. Gelvin S. B. (1994). Association of single – stranded transferred DNA from *Agrobacterium tumefaciens* with tobacco cells. Proc. Natl. Acad. Sci. U.S.A. 91, 2994–2998. 10.1073/pnas.91.8.2994 8159693 PMC43501

[B181] Zaenen I. Van Larabeke N. Teuchy H. Van Montagu M. Schell J. (1974). Supercoiled circular DNA in crown gall inducing *Agrobacterium* strains. J. Mol. Biol. 86, 109–127 10.1016/S0022-2836(74)80011-2 4854526

[B182] Zaltsman A. Krichevsky A. Loyter A. Citovsky V. (2010). *Agrobacterium* induces expression of a host F-box protein required for tumorigenicity. Cell Host Microbe 7, 197–209. 10.1016/j.chom.2010.02.009 20227663 PMC3427693

[B183] Zambryski P. Holsters M. Kruger K. Depicker A. Schell J. Van Montagu M. (1980). Tumor DNA structure in plant cells transformed by *A. tumefaciens*. Science 209, 1385–1391 10.1126/science.6251546 6251546

[B184] Zhan X. Jones D. A. Kerr A. (1990). The pTiC58 *tzs* gene promotes high-efficiency root induction by agropine strain 1855 of *Agrobacterium rhizogenes*. Plant Mol. Biol. 14, 785–792. 10.1007/BF00016511 2102856

[B185] Zhao J. Binns A. (2011). Characterization of the mmsAB-araD1 (gguABC) genes of *Agrobacterium tumefaciens*. J. Bacteriol. 193, 6586–6596. 10.1128/JB.05790-11 21984786 PMC3232879

[B186] Ziemienowicz A. Merkle T. Schoumacher F. Hohn B. Rossi L. (2001). Import of *Agrobacterium* T-DNA into plant nuclei: two distinct functions of VirD2 and VirE2 proteins. Plant Cell 13, 369–383. 10.1105/tpc.13.2.369 11226191 PMC102248

[B187] Zou S. B. Hersch S. J. Roy H. Wiggers J. B. Leung A. S. Buranyi S. (2012). Loss of elongation factor P disrupts bacterial outer membrane integrity. J. Bacteriol. 194, 413–425. 10.1128/JB.05864-11 22081389 PMC3256640

[B188] Zou S. B. Roy H. Ibba M. Navarre W. W. (2011). Elongation factor P mediates a novel post-transcriptional regulatory pathway critical for bacterial virulence. Virulence 2, 147–151. 10.4161/viru.2.2.15039 21317554 PMC3265757

[B189] Zupan J. R. Citovsky V. Zambryski P. (1996). *Agrobacterium* VirE2 protein mediates nuclear uptake of single-stranded DNA in plant cells. Proc. Natl. Acad. Sci. U.S.A. 93, 2392–2397. 10.1073/pnas.93.6.2392 8637884 PMC39807

